# AMPK signaling in the nucleus accumbens core mediates cue-induced reinstatement of cocaine seeking

**DOI:** 10.1038/s41598-017-01043-5

**Published:** 2017-04-21

**Authors:** Xue-Jiao Gao, Kai Yuan, Lu Cao, Wei Yan, Yi-Xiao Luo, Min Jian, Jian-Feng Liu, Qin Fang, Ji-Shi Wang, Ying Han, Jie Shi, Lin Lu

**Affiliations:** 1 National Institute on Drug Dependence and Beijing Key Laboratory of Drug Dependence, Peking University, Beijing, China; 2 Department of Pharmacology, School of Basic Medical Sciences, Peking University Health Science Center, Beijing, China; 3 Peking University Sixth Hospital, Peking University Institute of Mental Health, Key Laboratory of Mental Health, Ministry of Health (Peking University), National Clinical Research Center for Mental Disorders (Peking University Sixth Hospital), Beijing, China; 4 Peking-Tsinghua Center for Life Sciences and PKU-IDG/McGovern Institute for Brain Research, Peking University, Beijing, China; 5 Affiliated Hospital and School of Pharmacy of Guizhou Medical University, Guiyang, China; 6 Department of Pharmacy, Medical College, Hunan Normal University, Changsha, China; 7 Department of Pharmacology and Toxicology, University at Buffalo, State University of New York, Buffalo, NY USA

**Keywords:** Addiction, Addiction

## Abstract

Relapse to drug seeking can be caused by exposure to drug-associated cues, provoking drug craving even after prolonged abstinence. Recent studies demonstrated that AMP-activated protein kinase (AMPK) regulates neuronal morphology and membrane excitability in neurons. Here, we investigated the role of AMPK activity in the nucleus accumbens (NAc) in relapse to cocaine seeking. We found that exposure to drug-related cues reinstated cocaine-seeking behavior and increased AMPK and p70s6k phosphorylation in the NAc core but not shell. Augmenting AMPK activity by intra-NAc core infusions of the AMPK activator 5-amino-1-β-D-ribofuranosyl-imidazole-4-carboxamide (AICAR) or adenovirus expressing constitutively active subunits of AMPK decreased cue-induced reinstatement of cocaine seeking and inhibited the mammalian target of rapamycin complex 1 (mTORC1) and extracellular signal-regulated kinase 1/2 (ERK1/2) pathways. In contrast, inhibition of AMPK activity by intra-NAc core infusions of the AMPK inhibitor compound C or adenovirus expressing dominant-negative subunits of AMPK increased cue-induced reinstatement of cocaine seeking and enhanced mTORC1 and ERK1/2 activity. The regulation of AMPK activity in the NAc shell had no effect on cue-induced cocaine seeking. Altogether, these results indicate that AMPK activity in the NAc core is critical for the cue-induced reinstatement of cocaine seeking, which may be mediated by mTORC1 and ERK1/2 signaling.

## Introduction

Addiction is a chronic relapsing disorder, characterized by compulsive drug taking and seeking behaviors despite harmful consequences. Repeated exposure to addictive drugs leads to persistent alterations in genetic expression and synaptic and structural plasticity in limbic regions, such as the nucleus accumbens (NAc), and enhances the brain’s reactivity to drug-associated cues^1–3^. These changes are enduring and contribute to drug relapse even after long periods of abstinence^4–7^. Relapse to drug seeking has been widely assessed in models of reinstatement, which can be triggered by acute priming injections of drugs, drug-associated cues, and environmental stressors^8–11^. Cue-induced drug craving is a cardinal feature of addictive disorders and triggers relapse to drug use after prolonged abstinence^5^.

Adenosine monophosphate-activated protein kinase (AMPK) is a serine/threonine heterotrimeric protein kinase and pivotal regulator of cellular energy homeostasis in both the central nervous system and peripheral organs^12^. AMPK participates in the regulation of various physiological processes, including the cell cycle, cell polarity, cell growth, cell development, autophagy, and the life span^13–15^. The dysregulation of AMPK signaling is also associated with various pathological states, such as obesity and cancer^16, 17^. Hypothalamic AMPK activity controls food intake by altering the expression of anorexigenic or orexigenic neuropeptides, such as neuropeptide Y and melanin concentrating hormone^18–20^. These neuropeptides and their receptors play important modulatory roles in the rewarding and reinforcing effects of addictive drugs and reinstatement of drug seeking^21–24^. Recent studies also showed that AMPK regulates neuronal morphology and membrane excitability in neurons by acting on receptors and channels^25–27^, and AMPK dysregulation is involved in the process of neurodegeneration^28–30^. Both presynaptic and postsynaptic AMPK signaling regulates fasting-induced synaptic plasticity in agouti-related peptide (AgRP)-expressing neurons^31, 32^, which influences midbrain dopamine neuronal activity and behavioral responses to cocaine^33^. Our previous study showed that AMPK negatively regulated contextual fear memory formation in a mammalian target of rapamycin complex 1 (mTORC1)-dependent manner^34^. These studies indicate that AMPK is important under both physiological and pathological conditions.

The NAc is a key brain reward region that plays a critical role in cue-induced drug seeking behaviors^35–37^. In the present study, we investigated the role of AMPK signaling in the NAc in drug relapse using the cue-induced reinstatement model.

## Results

### Drug-related cue exposure induced the reinstatement of cocaine seeking and activated the AMPK and mTORC1 signaling pathways

**Figure 1:**
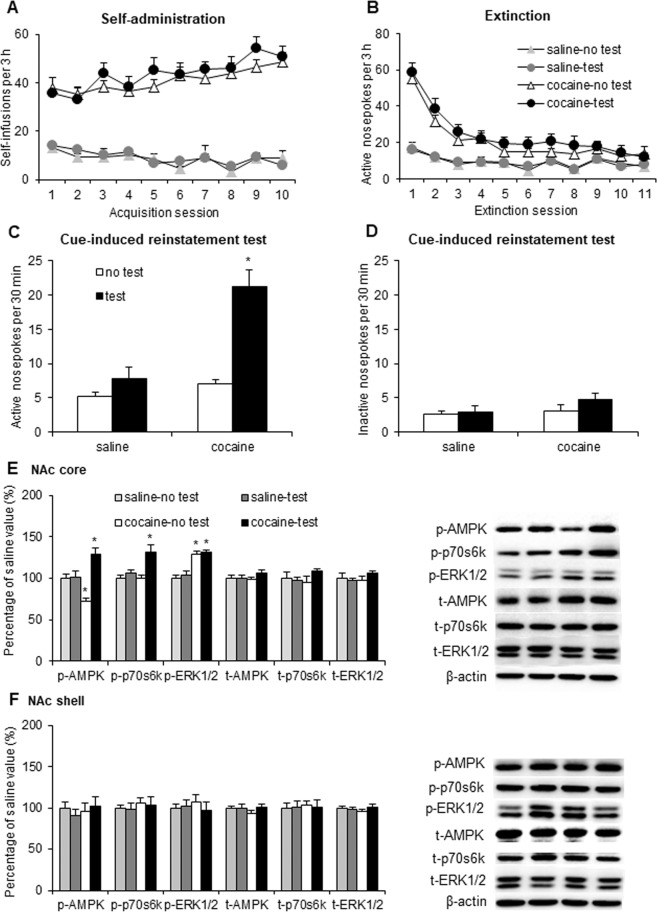
Exposure to cocaine-related cues reinstated cocaine-seeking behavior and altered the AMPK signaling pathway in the NAc core. (**A**) The number of self-infusions during daily 3-h sessions increased in the cocaine self-administration groups but not in the saline groups (n = 8–9 per group). (**B**) Responding during daily 3-h extinction sessions decreased in the cocaine groups. (**C**,**D**) Average active and inactive nosepoke responding during a 30-min session (reinstatement) in which either no cue was presented (i.e., another extinction session; no test) or cue-induced reinstatement was induced by presentation of cocaine-associated cues (test). (**E**,**F**) Percentage of phosphorylated AMPK, phosphorylated p70s6k, phosphorylated ERK1/2, total AMPK, total p70s6k, and total ERK1/2 in the NAc core and shell relative to the saline-no test group and representative Western blots (*n* = 8 per group). The data are expressed as mean ± SEM. *p < 0.05, compared with saline no test group.

We first tested the effect of exposure to cocaine-associated cues on cocaine seeking and AMPK/p70s6k/ERK activity in the NAc core and shell. Four groups of rats were used in a 2 (reward type: saline and cocaine) × 2 (reinstatement test: no test and test) factorial design. We trained the rats to self-administer intravenous cocaine for 10 days. The rats then underwent extinction for 11 days. The number of cocaine infusions during the training sessions and number of active nosepoke responses during the extinction sessions did not differ among groups (Fig. 1A,B). A two-way analysis of variance (ANOVA), with reward type and reinstatement test as the between-subjects factors, was performed to analyze active and inactive nosepoke responses in the cue-induced reinstatement tests. The ANOVA revealed significant effects of reward type (*F*_1,29_ = 25.739, *p* < 0.01; Fig. 1C) and reinstatement test (*F*_1,29_ = 31.016, *p* < 0.01; Fig. 1C) and a significant reinstatement test × reward type interaction (*F*_1,29_ = 14.697, *p* < 0.01; Fig. 1C) for responses on the active but not inactive (*p* > 0.05; Fig. 1D) nosepoke operandum. The *post hoc* analysis showed that the number of active nosepokes during the cue-induced reinstatement test after extinction from cocaine was higher than the other groups (Fig. 1C). For all of the groups, the number of nosepokes in the inactive operandum was very low, with no significant differences between groups (Fig. 1D).

The rats were decapitated after the cue-induced reinstatement test, and their brains were extracted for the subsequent determination of phosphorylated AMPK (p-AMPK; Thr172), phosphorylated p70s6k (p-p70s6k; Thr389), and phosphorylated ERK1/2 (p-ERK1/2; Thr202/Tyr204) in the NAc core and shell. The two-way ANOVA revealed significant reinstatement test × reward type interactions (p-AMPK: *F*_1,28_ = 23.466, *p* < 0.01; p-p70s6k: *F*_1,28_ = 6.518, *p* < 0.05; Fig. 1E) in the NAc core. The one-way ANOVA of p-ERK1/2 revealed an effect of experimental group (*F*_3,31_ = 30.272, *p* < 0.01; Fig. 1E) in the NAc core. The *post hoc* analysis showed that exposure to cocaine cues after extinction increased phosphorylated but not total AMPK, p70s6k, and ERK1/2 in the NAc core (Fig. 1E). The levels of p-AMPK decreased and the levels of p-ERK1/2 increased in the NAc core in rats that underwent the extinction test after cocaine self-administration and extinction training, compared with the saline-no test group (Fig. 1E). Dephosphorylated AMPK in the cocaine-no test group may be attributable to cocaine exposure or extinction training. No changes were observed in the NAc shell (*p* > 0.05; Fig. 1F). These results indicate that exposure to cocaine-associated cues increased AMPK and mTORC1 activity in the NAc core but not shell.

### AMPK activation in the NAc core attenuated the cue-induced reinstatement of cocaine seeking

**Figure 2:**
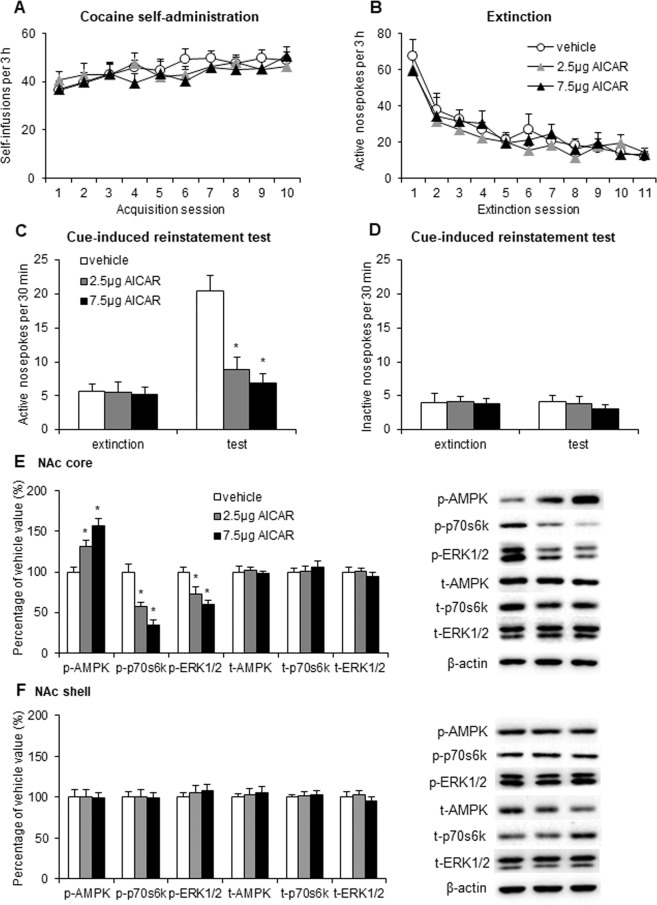
Activation of AMPK activity in the NAc core decreased the cue-induced reinstatement of cocaine seeking and inhibited mTORC1 and ERK1/2 signaling. (**A**) The number of self-infusions during daily 3-h sessions increased in the cocaine self-administration groups (*n* = 9 per group). (**B**) Responding during daily 3-h extinction sessions decreased in the cocaine groups. (**C**,**D**) Average active or inactive nosepoke responding during a 30-min cue-induced reinstatement session before which the rats received intra-NAc core infusions of vehicle or AICAR (2.5 or 7.5 μg/side). (**E**,**F**) Percentage of phosphorylated AMPK, phosphorylated p70s6k, phosphorylated ERK1/2, total AMPK, total p70s6k, and total ERK1/2 in the NAc core and shell relative to the vehicle group and representative Western blots (*n* = 9 per group). The data are expressed as mean ± SEM. *p < 0.05, compared with vehicle group.

We then tested the effects of AMPK activation on cocaine seeking after extinction. We trained the rats to self-administer intravenous cocaine for 10 days and then subjected them to extinction for 11 days. The rats received microinjections of vehicle (0.5 μl/side) or the AMPK activator 5-amino-1-β-D-ribofuranosyl-imidazole-4-carboxamide (AICAR; 2.5 or 7.5 μg/side) in the NAc core 30 min before the cue-induced reinstatement test. The number of cocaine infusions during the training sessions and active nosepoke responses during the extinction sessions did not differ among groups (Fig. 2A,B). The repeated-measures ANOVA revealed significant effects of cue exposure (*F*_1,48_ = 27.079, *p* < 0.01; Fig. 2C) and AICAR dose (*F*_2,48_ = 11.626, *p* < 0.01; Fig. 2C) and a significant cue exposure × AICAR dose interaction (*F*_2,48_ = 10.388, *p* < 0.01; Fig. 2C) for active nosepoke responses. The *post hoc* analysis showed that intra-NAc core infusions of AICAR decreased responding on the active nosepoke operandum in the reinstatement test (*p* < 0.01; Fig. 2C). AICAR infusions had no effect on inactive nosepoke responding in the reinstatement test (*p* > 0.05; Fig. 2D). The Western blot assays showed that intra-NAc core infusions of AICAR increased p-AMPK (*F*_2,26_ = 20.823, *p* < 0.01; Fig. 2E) and decreased p-p70s6k (*F*_2,26_ = 47.948, *p* < 0.01; Fig. 2E) and p-ERK1/2 (*F*_2,26_ = 12.887, *p* < 0.01; Fig. 2E) in the NAc core but not shell (*p* > 0.05; Fig. 2F).

These results indicate that AMPK activation in the NAc core decreased cue-induced cocaine seeking and inhibited mTORC1 and ERK1/2 signaling pathways.

### AMPK inhibition in the NAc core enhanced the cue-induced reinstatement of cocaine seeking

**Figure 3:**
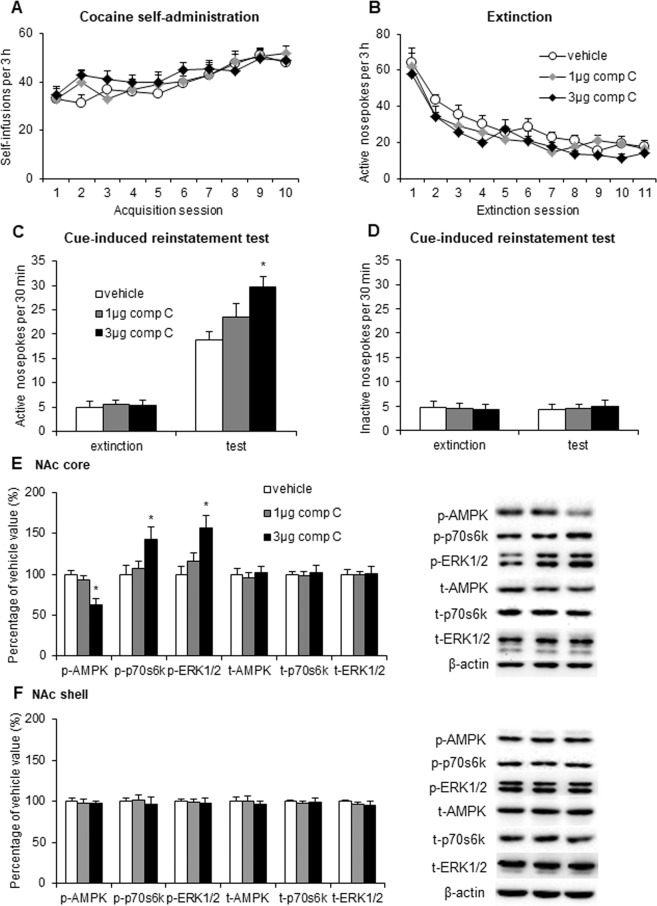
Inhibition of AMPK activity in the NAc core increased the cue-induced reinstatement of cocaine seeking and enhanced mTORC1 and ERK1/2 signaling. (**A**) The number of self-infusions during daily 3-h sessions increased in the cocaine self-administration groups (*n* = 9 per group). (**B**) Responding during daily 3-h extinction sessions decreased in the cocaine groups. (**C**,**D**) Average active and inactive nosepoke responding during a 30-min cue-induced reinstatement session before which the rats received intra-NAc core infusions of vehicle or compound C (1 or 3 μg/side). (**E**,**F**) Percentage of phosphorylated AMPK, phosphorylated p70s6k, phosphorylated ERK1/2, total AMPK, total p70s6k, and total ERK1/2 in the NAc core and shell relative to the vehicle group and representative Western blots (*n* = 9 per group). The data are expressed as mean ± SEM. *p < 0.05, compared with vehicle group.

We then tested the effects of AMPK inhibition on cocaine seeking after extinction. We trained the rats to self-administer intravenous cocaine for 10 days and then subjected them to extinction for 11 days. The rats received microinjections of vehicle (0.5 μl/side) or the AMPK inhibitor compound C (1 or 3 μg/side) in the NAc core 30 min before the cue-induced reinstatement test. The number of cocaine infusions during the training sessions and active nosepoke responses during the extinction sessions did not differ among groups (Fig. 3A,B). The repeated-measures ANOVA revealed significant effects of cue exposure (*F*_1,48_ = 182.088, *p* < 0.01; Fig. 3C) and compound C dose (*F*_2,48_ = 5.512, *p* < 0.01; Fig. 3C) and a significant cue exposure × compound C dose interaction (*F*_2,48_ = 4.761, *p* < 0.05; Fig. 3C) for active nosepoke responding. The *post hoc* analysis showed that intra-NAc core infusions of compound C increased responding on the active nosepoke operandum in the reinstatement test (Fig. 3C). Intra-NAc core infusions of compound C had no effect on inactive nosepoke responding in the reinstatement test (*p* > 0.05; Fig. 3D). The Western blot assays showed that intra-NAc core infusions of compound C decreased p-AMPK (*F*_2,26_ = 13.629, *p* < 0.01; Fig. 3E) and increased p-p70s6k (*F*_2,26_ = 5.149, *p* < 0.05; Fig. 3E) and p-ERK1/2 (*F*_2,26_ = 7.929, *p* < 0.01; Fig. 3E) in the NAc core but not shell (*p* > 0.05; Fig. 3F).

These results indicate that AMPK inhibition in the NAc core enhanced cue-induced cocaine seeking and increased mTORC1 and ERK1/2 activity.

### Regulation of AMPK activity in the NAc shell had no effect on cue-induced cocaine seeking

**Figure 4:**
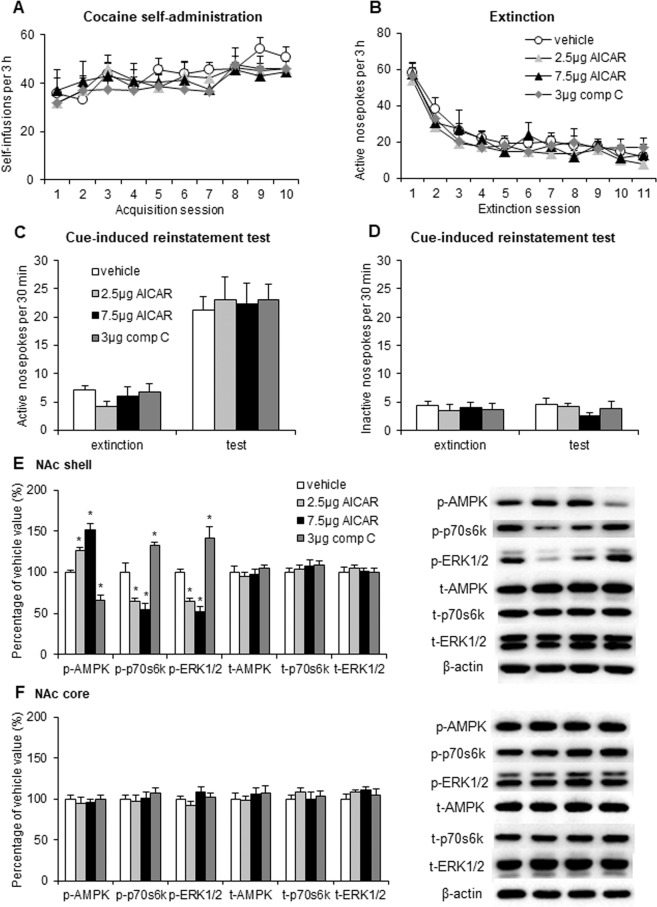
Regulation of AMPK activity in the NAc shell did not alter cue-induced cocaine seeking. (**A**) The number of self-infusions during daily 3-h sessions increased in the cocaine self-administration groups (*n* = 7–9 per group). (**B**) Responding during daily 3-h extinction sessions decreased in the cocaine groups. (**C**,**D**) Average active and inactive nosepoke responding during a 30-min cue-induced reinstatement session before which the rats received intra-NAc shell infusions of vehicle or AICAR (2.5 or 7.5 μg/side) or compound C (3 μg/side). (**E**,**F**) Percentage of phosphorylated AMPK, phosphorylated p70s6k, phosphorylated ERK1/2, total AMPK, total p70s6k, and total ERK1/2 in the NAc shell and core relative to the vehicle group and representative Western blots (*n* = 7 per group). The data are expressed as mean ± SEM. *p < 0.05, compared with vehicle group.

We next tested the effects of pharmacologically modulating AMPK activity in the NAc shell on cocaine seeking after extinction. We trained the rats to self-administer intravenous cocaine for 10 days and then subjected them to extinction for 11 days. The rats received microinjections of vehicle (0.5 μl/side), AICAR (2.5 or 7.5 μg/side), or compound C (3 μg/side) in the NAc shell 30 min before the cue-induced reinstatement test. The number of cocaine infusions during the training sessions and active nosepoke responses during the extinction sessions did not differ among groups (Fig. 4A,B). The pharmacological modulation of AMPK activity in the NAc shell had no significant effects on active (Fig. 4C) or inactive (Fig. 4D) nosepoke responses in the reinstatement test. Nucleus accumbens shell infusions of AICAR and compound C altered p-AMPK (*F*_3,27_ = 55.353, *p* < 0.01; Fig. 4E), p-p70s6k (*F*_3,27_ = 59.001, *p* < 0.01; Fig. 4E), and p-ERK1/2 (*F*_3,27_ = 24.373, *p* < 0.01; Fig. 4E) in the NAc shell but not core (all *p* > 0.05; Fig. 4F).

### Regulation of AMPK activity in the NAc core by adenovirus-mediated gene transfer altered cue-induced cocaine seeking

**Figure 5:**
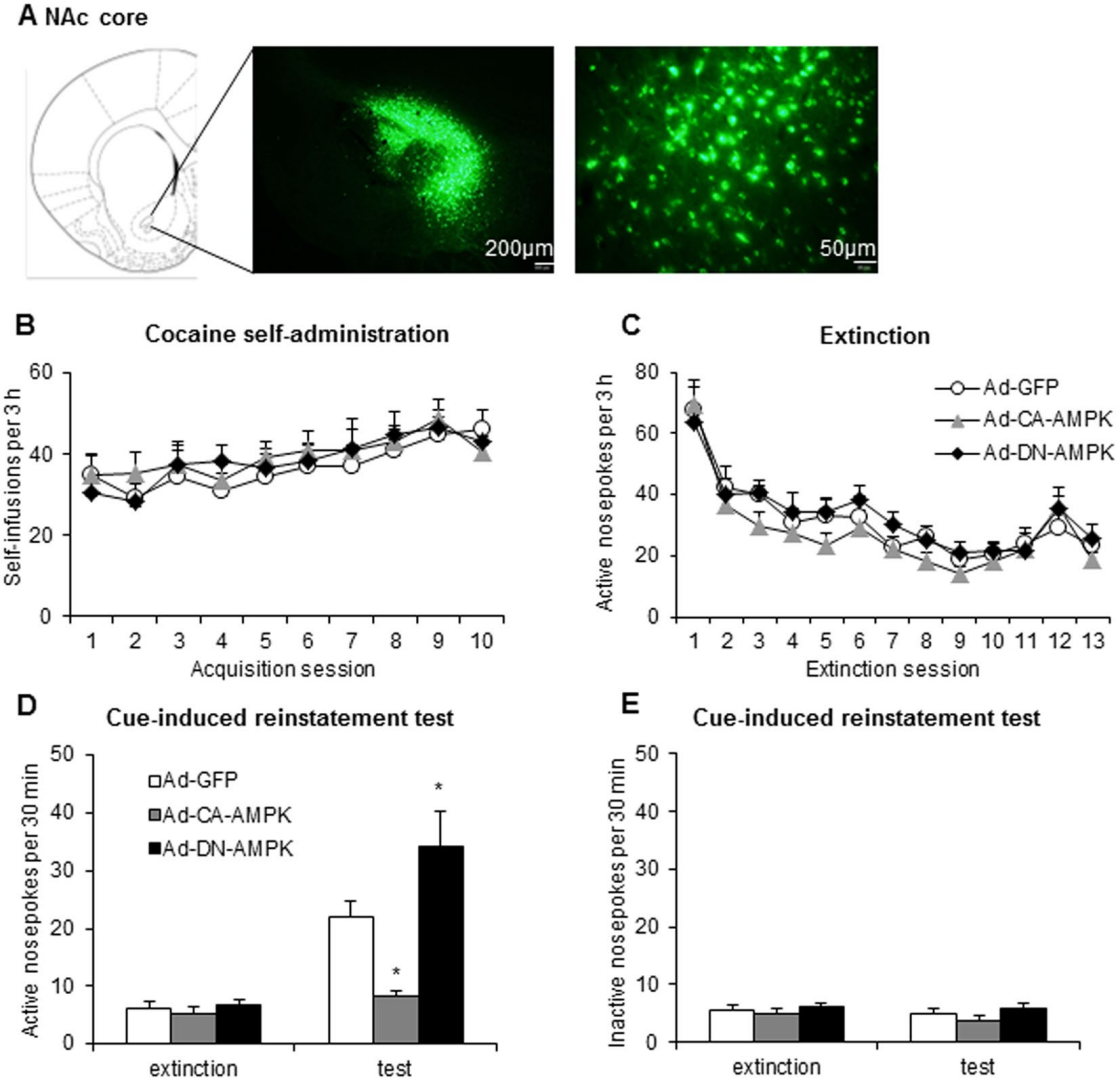
Regulation of AMPK activity in the NAc core by adenovirus-mediated gene transfer altered cue-induced cocaine seeking. (**A**) Representative photographs of the injection sites and coronal brain sections in the NAc core. The figure shows representative micrographs of adenovirus vector-mediated enhanced green fluorescent protein (eGFP; green) after microinjections in the NAc core. Scale bars = 200 μm (low-magnification images) and 50 μm (high-magnification images). (**B**) The number of self-infusions during daily 3-h sessions increased in the cocaine self-administration groups (*n* = 10 per group). (**C**) Responding during daily 3-h extinction sessions decreased in the cocaine groups. (**D**,**E**) Average active and inactive nosepoke responding during a 30-min cue-induced reinstatement session before which the rats received intra-NAc core infusions of an adenovirus that expressed constitutively active (CA) or dominant-negative (DN) subunits of AMPK. The data are expressed as mean ± SEM. *p < 0.05, compared with Ad-GFP group.

After finding that the pharmacological modulation of AMPK activity in the NAc core regulated the cue-induced reinstatement of cocaine seeking, we further confirmed the effects of modulating AMPK activity on drug relapse by expressing a constitutively active (CA) subunit of AMPK (T172D mutation in the α2 subunit of AMPK) or dominant-negative (DN) subunit of AMPK (K45R mutation in the α2 subunit of AMPK) in the NAc core using recombinant adenovirus. We first examined the efficiency of modulating AMPK activity by adenoviral transfer in the NAc core (Fig. 5A).

We trained the rats to self-administer intravenous cocaine for 10 days and then subjected them to extinction for 13 days. Three days before the cue-induced reinstatement test, the rats received infusions of Ad-GFP, Ad-CA-AMPK, or Ad-DN-AMPK in the NAc core. The number of cocaine infusions during the training sessions and active nosepoke responses during the extinction sessions did not differ among groups (Fig. 5B,C). The repeated-measures ANOVA revealed significant effects of cue exposure (*F*_1,54_ = 42.045, *p* < 0.01; Fig. 5D) and vector (*F*_2,54_ = 10.896, *p* < 0.01; Fig. 5D) and a significant cue exposure × vector interaction (*F*_2,54_ = 8.761, *p* < 0.01; Fig. 5D) for active nosepoke responding. The *post hoc* analysis showed that increasing AMPK activity by infusing Ad-CA-AMPK in the intra-NAc core decreased responding on the active nosepoke operandum, whereas decreasing AMPK activity by infusing Ad-DN-AMPK in the intra-NAc core increased responding on the active nosepoke operandum in the reinstatement test (Fig. 5D). The regulation of AMPK activity in the NAc core by an adenovirus that expressed CA-AMPK or DA-AMPK subunits had no effect on inactive nosepoke responding in the reinstatement test (*p* > 0.05; Fig. 5E).

## Discussion

In the present study, we investigated the role of AMPK activity in the NAc in the cue-induced relapse of cocaine seeking. We found that exposure to cocaine-associated cues induced the phosphorylation of AMPK and p70s6k in the NAc core but not shell. Using pharmacological methods and adenovirus gene transfer to bidirectionally regulate AMPK activity, we found that increasing AMPK activity in the NAc core inhibited the cue-induced reinstatement of cocaine seeking, and decreasing AMPK activity enhanced cue-induced cocaine seeking. These behavioral changes were associated with alterations in the phosphorylation of AMPK, p70s6k, and ERK1/2 in the NAc core. Altogether, these results indicate that AMPK activity in the NAc core regulated cue-induced relapse to cocaine seeking, and these effects may be mediated by mTORC1 and ERK1/2 signaling.

The NAc core and shell differ in their projection patterns and functions. The NAc core receives afferents predominantly from the amygdala and dorsal medial prefrontal cortex and plays an important role in cue-conditioned motivated behaviors, including drug seeking^38–41^. The NAc shell mainly receives afferents from the hippocampus and ventral medial prefrontal cortex and is involved in context-induced relapse to drug seeking^42, 43^. In the present study, we found that exposure to cocaine-associated cues induced AMPK phosphorylation in the NAc core but not shell. Furthermore, modulating AMPK activity in the NAc core but not shell altered cue-induced cocaine seeking. These results indicate that the effects of AMPK activity on the relapse to cocaine seeking were anatomically specific.

AMPK is a member of serine/threonine kinases and well known as a master regulator of cellular and organismal metabolism. AMPK is also highly expressed in the central nervous system and regulates neural differentiation and brain development^44, 45^. Hypothalamic AMPK activity regulates AgRP neuronal activity and controls food intake^18, 31, 32^. AMPK dysfunction contributes to the pathogenesis of several neurodegenerative disorders, including Alzheimer’s disease, Huntington’s disease, and amyotrophic lateral sclerosis^28–30^. Our previous study also found that AMPK in the CA1 negatively regulated fear memory formation through mTORC1 signaling^34^. In the present study, we found that augmenting AMPK activity in the NAc core decreased cue-induced relapse to cocaine seeking, and decreasing AMPK activity enhanced cue-induced relapse to cocaine seeking. These results indicate that AMPK activity in the NAc core plays a critical role in cocaine seeking behaviors that are triggered by drug-associated cues.

As a multifunctional enzyme, AMPK can directly phosphorylate the tumor suppressor TSC2 and raptor, thus suppressing the mTORC1 pathway and regulating cell growth and autophagy^14^. In the present study, we found that exposure to cocaine-associated cues increased the phosphorylation of both AMPK and p70s6k in the NAc core. A compensatory mechanism may coordinate the balance between AMPK and mTORC1 signaling in response to cocaine-associated cues that induce relapse. AMPK can also phosphorylate B-Raf proto-oncogene serine/threonine-protein kinase at Ser729, promote its association with the 14-3-3 signaling adaptor, and prevent the paradoxical activation of mitogen-activated protein kinase kinase (MEK)-ERK signaling in keratinocytes^46^. Inhibitory cross-talk between the AMPK and ERK pathways mediates endoplasmic reticulum stress-induced insulin resistance and cell proliferation^47, 48^. Consistent with these studies, we found that AMPK activation decreased p70s6k and ERK1/2 phosphorylation, while AMPK inhibition increased p70s6k and ERK1/2 phosphorylation. We speculate that the activation of AMPK during cue-induced reinstatement test may be an important compensatory response that decreases cocaine craving. When pharmacologically modulating AMPK activity in the NAc, AICAR or compound C may act on downstream mTORC1 and ERK1/2 pathways and alter cue-induced relapse to cocaine seeking.

ERK inhibition blocked the neuronal activity-induced activation of mTORC1 signaling, such as phosphorylation of the translation factors p70s6k, 4EBP1, and ribosomal protein S6^49^. There is a convergence of AMPK signaling with mTORC1 and ERK1/2 signaling in growth and metabolic control^14^. Interactions between ERK and mTOR signaling play an important role in protein synthesis, long-term synaptic plasticity, and memory^50–52^. The mTOR and ERK signaling pathways also play important roles in cue-directed cocaine seeking^53, 54^. In the present study, we found that the modulation of AMPK activity in the NAc core either increased or decreased the cue-induced reinstatement of cocaine seeking and consistently altered the phosphorylation of p70s6k and ERK1/2 locally. These results indicate that the mTORC1 and ERK1/2 signaling pathways may mediate the effects of AMPK activity on cue-induced cocaine seeking.

In summary, we found that AMPK activity in the NAc core plays a critical role in cue-induced relapse to cocaine seeking. The mTORC1 and ERK1/2 signaling pathways may coordinate with AMPK signaling to regulate cocaine seeking. Targeting AMPK signaling may be a practical therapeutic strategy for preventing relapse.

## Methods

### Subjects

One hundred and forty-eight male Sprague Dawley rats, weighing 240–260 g, were obtained from the Laboratory Animal Center, Peking University Health Science Center. They were housed five per cage in a temperature- (23 °C ± 2 °C) and humidity- (50% ± 5%) controlled animal facility with *ad libitum* access to food and water^55, 56^. They were kept on a reverse 12 h/12 h light/dark cycle. The behavioral experiments were conducted during the dark phase of the cycle. All of the experiments were performed according to the National Institutes of Health Guide for the Care and Use of Laboratory Animals and were approved by the Biomedical Ethics Committee on animal use and protection of Peking University.

### Intracranial and intravenous surgery

Rats (weighing 280–300 g when surgery began) were anesthetized with sodium pentobarbital (50 mg/kg, i.p.). Catheters were inserted into the right jugular vein with the tip terminating at the opening of the right atrium as described previously^53, 57, 58^. Guide cannulae (23 gauge; Plastics One, Roanoke, VA, USA) were bilaterally implanted 1 mm above the NAc core or shell. The cannulae were placed at a 16° angle toward the midline to avoid penetration of the lateral ventricle. The coordinates for the NAc core were the following: anterior/posterior, +1.5 mm; medial/lateral, ±3.8 mm; dorsal/ventral, −6.2 mm. The coordinates for the NAc shell were the following: anterior/posterior, +1.8 mm; medial/lateral, ±3.2 mm; dorsal/ventral, −6.6 mm^59–61^. The cannulae were anchored to the skull with stainless-steel screws and dental cement. A stainless-steel stylet blocker was inserted into each cannula to maintain patency and prevent infection. The rats were allowed to recover for 7 days after surgery.

### Design, construction, and validation of adenoviral vectors for AMPK subunits

Constitutively active AMPKα2 cDNA constructs (T172D mutant) and DN AMPKα2 cDNA constructs (K45R mutant) were designed and constructed according to previous studies^34, 62, 63^. The CA-AMPK construct encodes residues 1–312 of AMPKα2 mutated on the threonine 172 residue to an aspartic acid (T172D). The DN-AMPK construct contains full-length AMPKα2 mutated on the lysine 45 residue to arginine (K45R). Appropriate mutagenesis was validated by sequencing. All AMPK cDNAs were subcloned into a pHBAd-CMV vector. All of the vectors contained the enhanced green fluorescence protein (eGFP) coding sequence (Hanbio Co. LTD).

### Intracranial injections of viruses and drugs

The AMPK activator AICAR was purchased from Toronto Research Chemicals, and the AMPK inhibitor compound C was purchased from Sigma (St. Louis, MO, USA). Compound C was dissolved in vehicle solution that contained 80% sterile saline, 10% dimethylsulfoxide (DMSO), and 10% cremophore EL (Sigma-Aldrich). AICAR was dissolved in saline. All of the drugs were freshly prepared before administration. The doses of the drugs were based on previous studies^29, 34, 64^. The infusion volume for all of the drugs was 0.5 μl. The drugs were infused bilaterally in the NAc core or shell using Hamilton syringes that were connected to 30-gauge injectors (Plastics One) that reached 1 mm below the guide cannula. The infusions occurred over 1 min, and the injection needle was kept in place for an additional 1 min to allow for drug diffusion^53^.

The experimental procedures that were used for the virus injections were based on previous studies^18, 34, 65^. The rats were anesthetized with sodium pentobarbital. The adenoviruses (1 × 10^11^ pfu/ml) were delivered bilaterally over 10 min at an infusion rate of 0.05 μl/min (total volume, 0.5 μl per side) using Hamilton syringes that were connected to 30-gauge injectors (Plastics One) that reached 1 mm below the guide cannula. The injectors were left in place for an additional 5 min to allow diffusion.

### Cocaine self-administration training

The procedures for cocaine self-administration training were based on previous studies with minor modifications^58, 66–68^. The chambers (AniLab Software & Instruments, Ningbo, China) were equipped with two nosepoke operandi (ENV-114M; Med Associates) located 9 cm above the floor of the chambers. Nosepokes in one (active) operandum led to cocaine infusions that were accompanied by a 5 s tone-light cue. Nosepokes in the other (inactive) operandum were also recorded but had no consequences. The rats were trained to self-administer intravenous cocaine hydrochloride (0.75 mg/kg/infusion) for 10 days, with three daily 1 h sessions that were separated by 5 min. The sessions began at the onset of the dark cycle. Each injection was accompanied by illumination of a cue light above the active nosepoke operandum, followed by an additional 40 s timeout period when the cue and house lights were extinguished and nosepoke responses had no programmed consequence. Each session began with illumination of a houselight that remained on for the entire session. The number of cocaine infusions was limited to 20 per hour. At the end of the training phase, the groups in the different experimental conditions were matched for their cocaine intake during training.

### Extinction training

During extinction, the stimulus light above the active nosepoke operandum was not illuminated. Responding on either nosepoke operandum had no programmed consequences (i.e., no cocaine infusion and no conditioned tone-light cue). The rats underwent extinction training until responding at the active nosepoke operandum decreased to below 20% of the mean responding during the last 3 days of cocaine self-administration for at least 2 consecutive days.

### Cue-induced reinstatement test

Once responding on the active nosepoke operandum was successfully extinguished according to the criteria described above, reinstatement testing commenced. The testing conditions were the same as during training, with the exception that active nosepokes were not reinforced with cocaine. Each session began with illumination of the house light, which remained on for the entire session. Nosepoke responding during the test sessions resulted in contingent presentations of the tone-light cue that was previously paired with cocaine infusions.

### Tissue sample preparation

The procedures were based on our previous studies^65, 69–71^. After decapitation, the brains were rapidly extracted and frozen in −60 °C N-hexane. The brains were then transferred to a −80 °C freezer. Bilateral tissue punches (16 gauge) of the NAc core and shell were placed in 1.5 ml microtubes that contained ice-cold radioimmunoprecipitation assay (RIPA) lysis buffer (Beyotime Biotechnology, Jiangsu, China). After homogenization by an electrical disperser (Wiggenhauser, Sdn Bhd), the homogenate was centrifuged at 10,000× *g* for 10 min at 4 °C. All of the above procedures were performed under low temperature (0–4 °C). The protein concentrations of the samples were determined using the bichinconinic acid assay (Beyotime Biotechnology, Jiangsu, China). The samples were further diluted in RIPA lysis buffer to equalize the protein concentrations. Four times loading buffer (16% glycerol, 20% β-mercaptoethanol, 2% sodium dodecyl sulfate [SDS], and 0.05% bromophenol blue) was added to each sample (3:1, sample:loading buffer) before being boiled for 5 min.

### Western blot

Equal amounts of protein (10–20 μg) for each sample were loaded onto SDS-polyacrylamide gel electrophoresis gels (10% acrylamide, 0.27% *N*,*N*’-methylenebisacryalamide resolving gel) for approximately 40 min at 80 V in stacking gel and approximately 1 h at 120 V in resolving gel. Proteins were electrophoretically transferred to Immobilon-P transfer membranes (Millipore, Bedford, MA, USA) at 250 mA for 2.5 h. Membranes were blocked with blocking buffer (5% bovine serum albumin [BSA] in TBST) for 2 h at room temperature. They were then incubated overnight at 4 °C with anti-phospho-AMPK antibody, anti-AMPK antibody, anti-phospho-p70s6k antibody, anti-p70s6k antibody, anti-phospho-ERK1/2 antibody, anti-ERK1/2 antibody (1:1000; Cell Signaling Technology, Danvers, MA, USA), or anti-β-actin antibody (1:1000; Santa Cruz Biotechnology, Santa Cruz, CA, USA) in TBST plus 5% BSA. After three 5-min washes in TBST buffer, the blots were incubated for 45 min at room temperature on a shaker with the corresponding horseradish peroxidase-conjugated secondary antibody (goat anti-mouse IgG for β-actin and goat anti-rabbit IgG for the others; 1:5000; Santa Cruz Biotechnology, Santa Cruz, CA, USA). The blots were washed three times for 5 min each in TBST, and immunostaining was visualized with a layer of Super Signal Enhanced chemiluminescence substrate (Detection Reagents 1 and 2, 1:1 ratio, Pierce Biotechnology, Rockford, IL, USA). The immunoblots were then screened using the ChemiDoc MP System (BioRad, Hercules, CA, USA) for 5–60 s. Band intensities were quantified using Quantity One 4.4.0 software (BioRad, Hercules, CA, USA).

### Histology

After the behavioral experiments, the rats were anesthetized with sodium pentobarbital (100 mg/kg, i.p.) and perfused with 0.01 mol/L phosphate-buffered saline (PBS) and 4% paraformaldehyde, pH 7.4. The brains were then extracted and postfixed in 4% paraformaldehyde for 24 h. Subsequently, the brains were cryoprotected in 30% sucrose in 0.2 mol/L phosphate buffer.

The cannula placements were assessed using Nissl staining with a section thickness of 40 μm under light microscopy. Rats with misplaced cannulae were excluded from the statistical analysis. The brains were coronally sectioned at 20 μm using a sliding microtome, and brain slices were examined for eGFP expression in adenoviral vector-injected rats using an Olympus BX53 fluorescent microscope^65, 72^. Representative images were captured at the same time.

### Statistical analysis

The data are expressed as mean ± SEM. The statistical analyses were performed using two-way ANOVA, repeat-measures ANOVA, or one-way ANOVA with appropriate between- and within-subject factors for each experiment (see Results section). *Post hoc* analyses of significant effects in the ANOVAs were performed using the Tukey test. Values of *p* < 0.05 were considered statistically significant.

## Supplementary information

Supplementary Information
