# Author Correction: AMPK signaling in the nucleus accumbens core mediates cue-induced reinstatement of cocaine seeking

**DOI:** 10.1038/s41598-020-61259-w

**Published:** 2020-03-06

**Authors:** Xue-Jiao Gao, Kai Yuan, Lu Cao, Wei Yan, Yi-Xiao Luo, Min Jian, Jian-Feng Liu, Qin Fang, Ji-Shi Wang, Ying Han, Jie Shi, Lin Lu

**Affiliations:** 1 National Institute on Drug Dependence and Beijing Key Laboratory of Drug Dependence, Peking University, Beijing, China; 2 Department of Pharmacology, School of Basic Medical Sciences, Peking University Health Science Center, Beijing, China; 3 Peking University Sixth Hospital, Peking University Institute of Mental Health, Key Laboratory of Mental Health, Ministry of Health (Peking University), National Clinical Research Center for Mental Disorders (Peking University Sixth Hospital), Beijing, China; 4 Peking-Tsinghua Center for Life Sciences and PKU-IDG/McGovern Institute for Brain Research, Peking University, Beijing, China; 5 Affiliated Hospital and School of Pharmacy of Guizhou Medical University, Guiyang, China; 6 Department of Pharmacy, Medical College, Hunan Normal University, Changsha, China; 7 Department of Pharmacology and Toxicology, University at Buffalo, State University of New York, Buffalo, NY USA

Correction to: *Scientific Reports* 10.1038/s41598-017-01043-5, published online 21 April 2017

The original version of this Article contained errors.

During the figure assembly some of the representative blots included in Figures 1, 2, 3, and 4 were duplicated or inappropriately cropped. The data for the following samples was incorrect: in Figure 1E image for t-AMPK; in Figure 2E image for t-p70s6k; in Figure 2F images for t-AMPK, t-p70s6k, and beta-actin; in Figure 3E images for t-p70s6k, t-ERK 1/2, and beta-actin; in Figure 3F images for p-AMPK, p-p70s6k, t-AMPK, t-p70s6k, t-ERK 1/2, beta-actin; and in Figure 4F image for p-p70s6k.

The original Figures 1, 2, 3, and 4 are included below.

**Figure 1:**
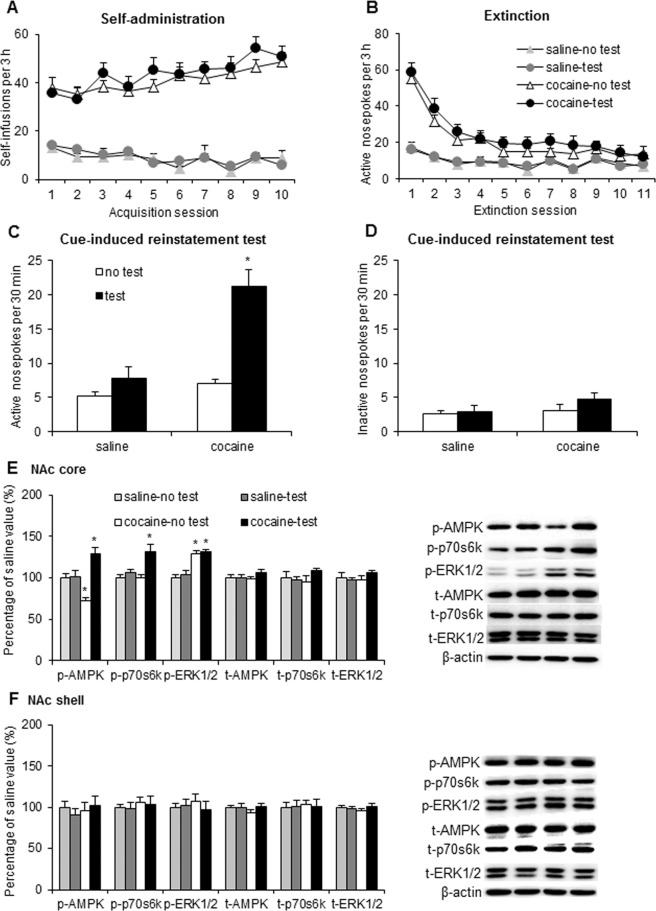
.

**Figure 2:**
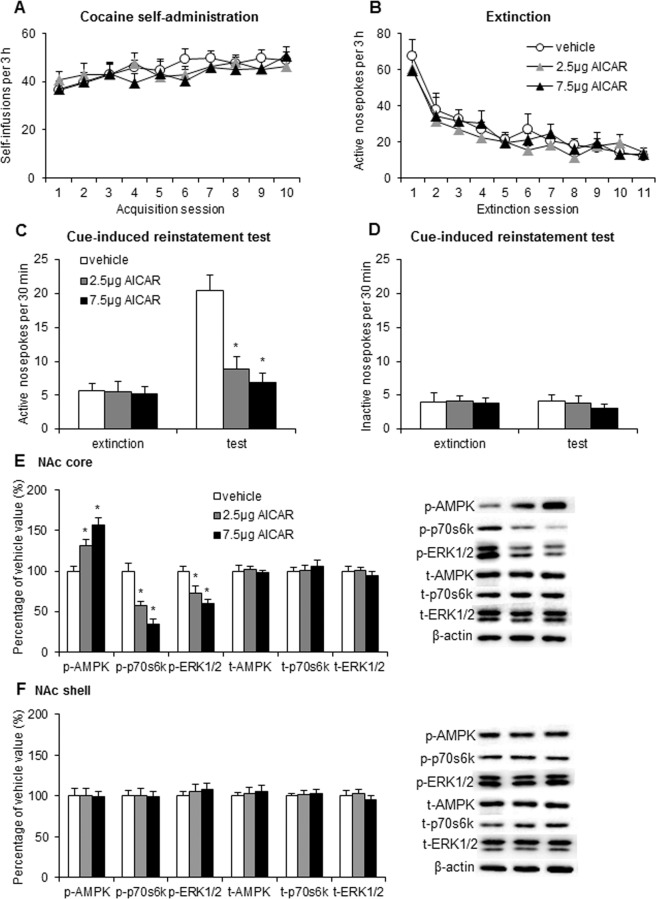
.

**Figure 3:**
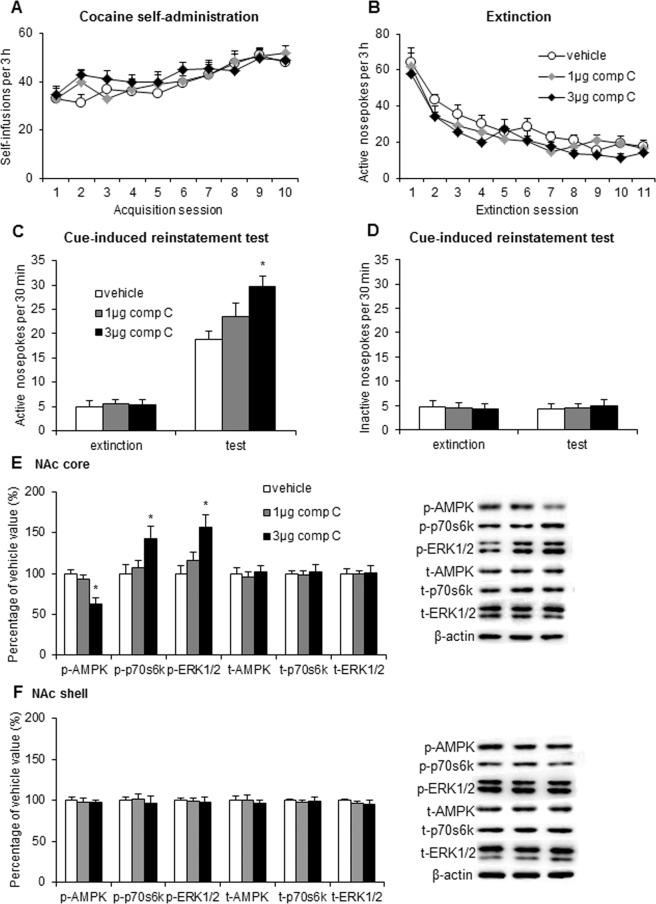
.

**Figure 4:**
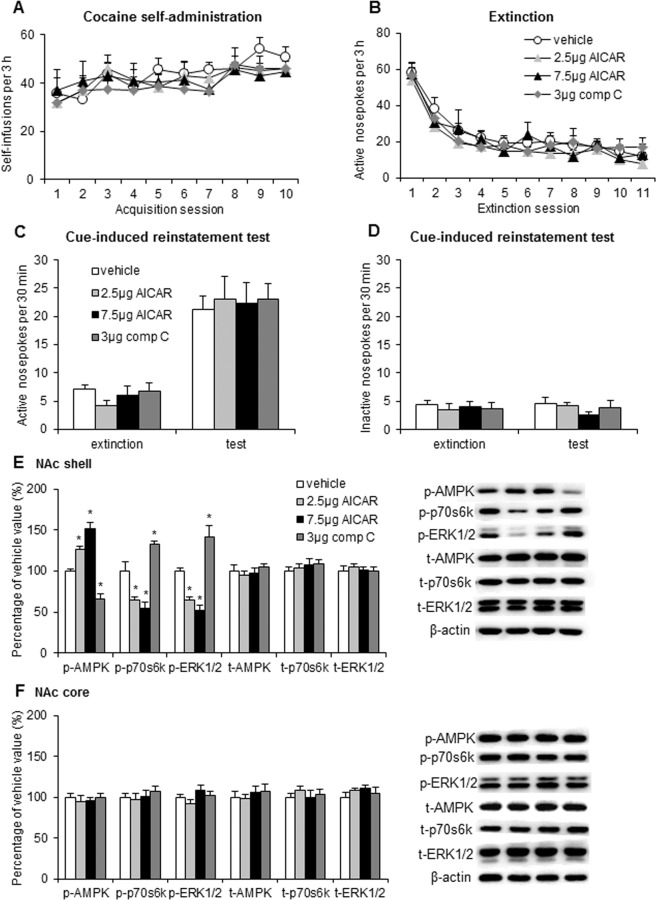
.

For transparency, the authors now also provide images of full length membranes for samples and all replicates. These are included in the new Supplementary File. Sections of the images used in the main figures are highlighted.

These errors have now been corrected in the PDF and HTML versions of the Article. The corrections do not affect the conclusions of the Article.

