# Supplementary material for: AMPK signaling in the nucleus accumbens core mediates cue-induced reinstatement of cocaine seeking
Source: Sci Rep. 2017 Apr 21;7:1038. doi: 10.1038/s41598-017-01043-5 (PMC5430902; doi:10.1038/s41598-017-01043-5)
Supplement: Supplementary file 1 — Supplementary Information [file 41598_2017_1043_MOESM1_ESM.pdf]

Supplementary Information

AMPK signaling in the nucleus accumbens core mediates cue-induced reinstatement of cocaine seeking

Xue-Jiao Gao, Kai Yuan, Lu Cao, Wei Yan, Yi-Xiao Luo, Min Jian, Jian-Feng Liu, Qin Fang, Ji-Shi Wang, Ying Han, Jie Shi, Lin Lu

A

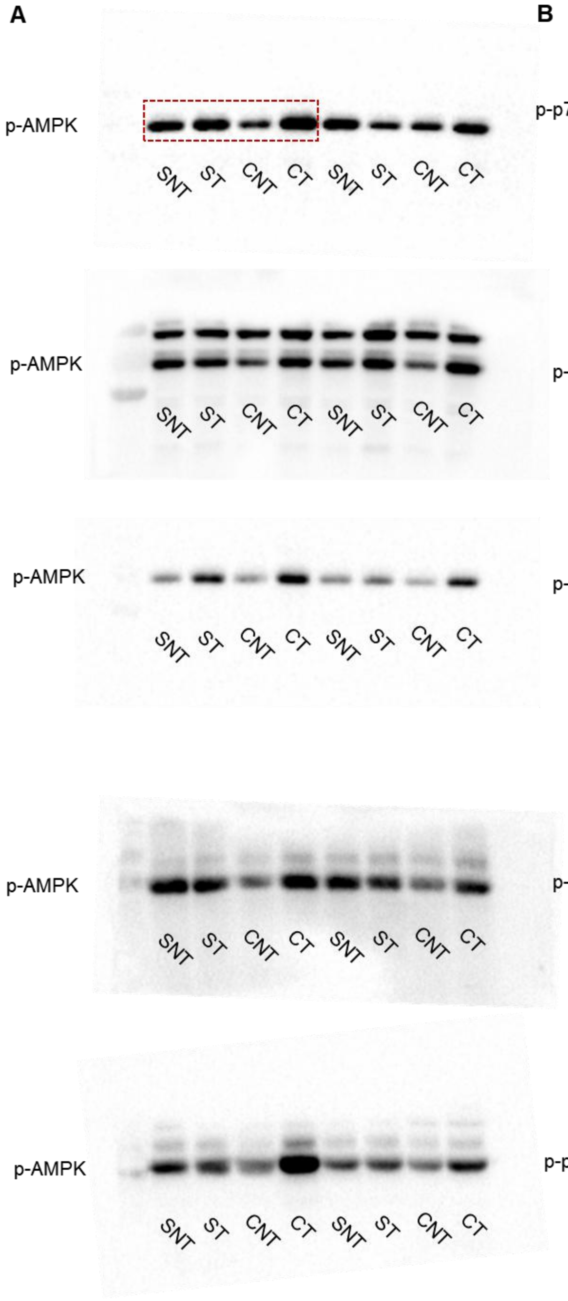
p-AMPK
SNT ST CNT CT SNT ST CNT CT
p-AMPK
SNT ST CNT CT SNT ST CNT CT
p-AMPK
SNT ST CNT CT SNT ST CNT CT
p-AMPK
SNT ST CNT CT SNT ST CNT CT
p-AMPK
SNT ST CNT CT SNT ST CNT CT

B

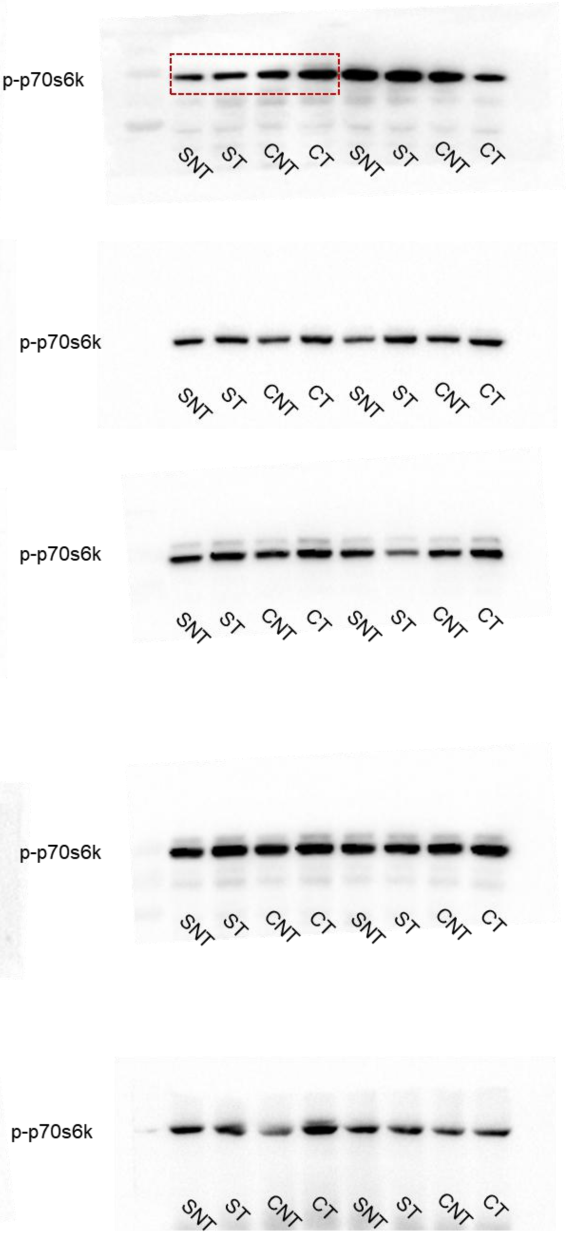
p-p70s6k
SNT ST CNT CT SNT ST CNT CT
p-p70s6k
SNT ST CNT CT SNT ST CNT CT
p-p70s6k
SNT ST CNT CT SNT ST CNT CT
p-p70s6k
SNT ST CNT CT SNT ST CNT CT
p-p70s6k
SNT ST CNT CT SNT ST CNT CT

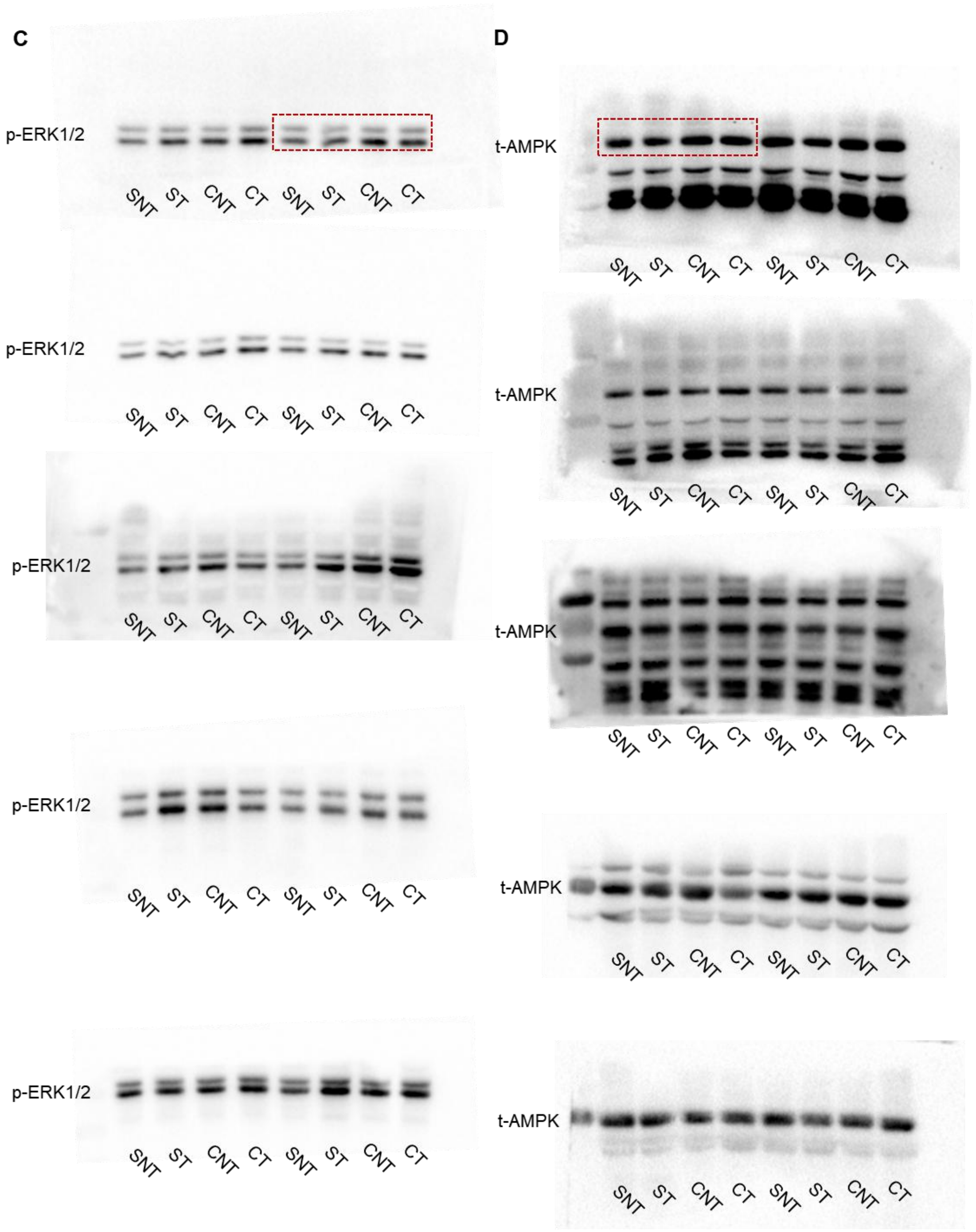
C
p-ERK1/2
SNT ST CNT CT SNT ST CNT CT
p-ERK1/2
SNT ST CNT CT SNT ST CNT CT
p-ERK1/2
SNT ST CNT CT SNT ST CNT CT
p-ERK1/2
SNT ST CNT CT SNT ST CNT CT
p-ERK1/2
SNT ST CNT CT SNT ST CNT CT
D
t-AMPK
SNT ST CNT CT SNT ST CNT CT
t-AMPK
SNT ST CNT CT SNT ST CNT CT
t-AMPK
SNT ST CNT CT SNT ST CNT CT
t-AMPK
SNT ST CNT CT SNT ST CNT CT
t-AMPK
SNT ST CNT CT SNT ST CNT CT

E

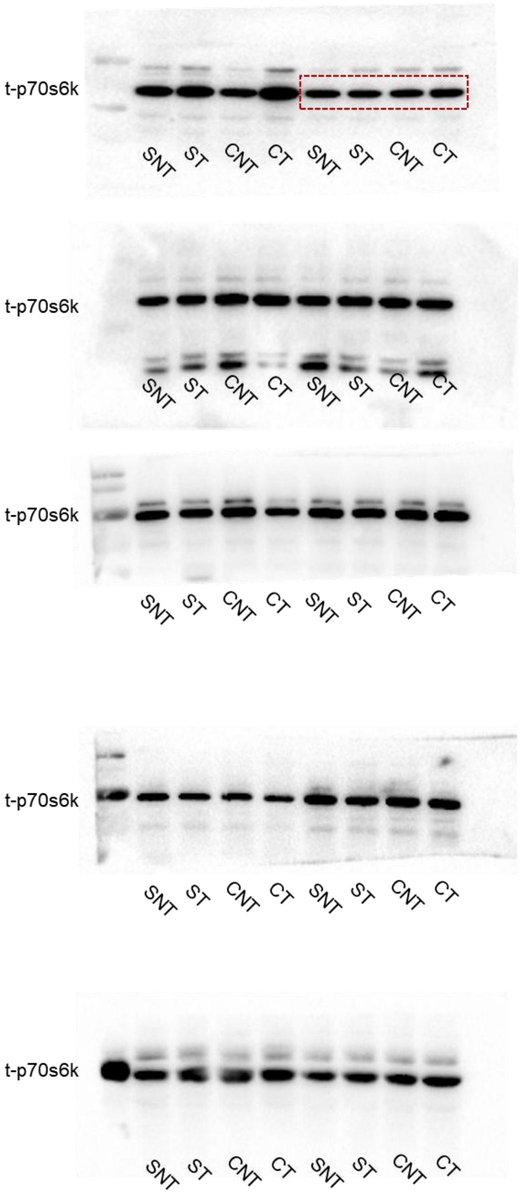
t-p70s6k
SNT ST CNT CT SNT ST CNT CT
t-p70s6k
SNT ST CNT CT SNT ST CNT CT
t-p70s6k
SNT ST CNT CT SNT ST CNT CT
t-p70s6k
SNT ST CNT CT SNT ST CNT CT
t-p70s6k
SNT ST CNT CT SNT ST CNT CT

F

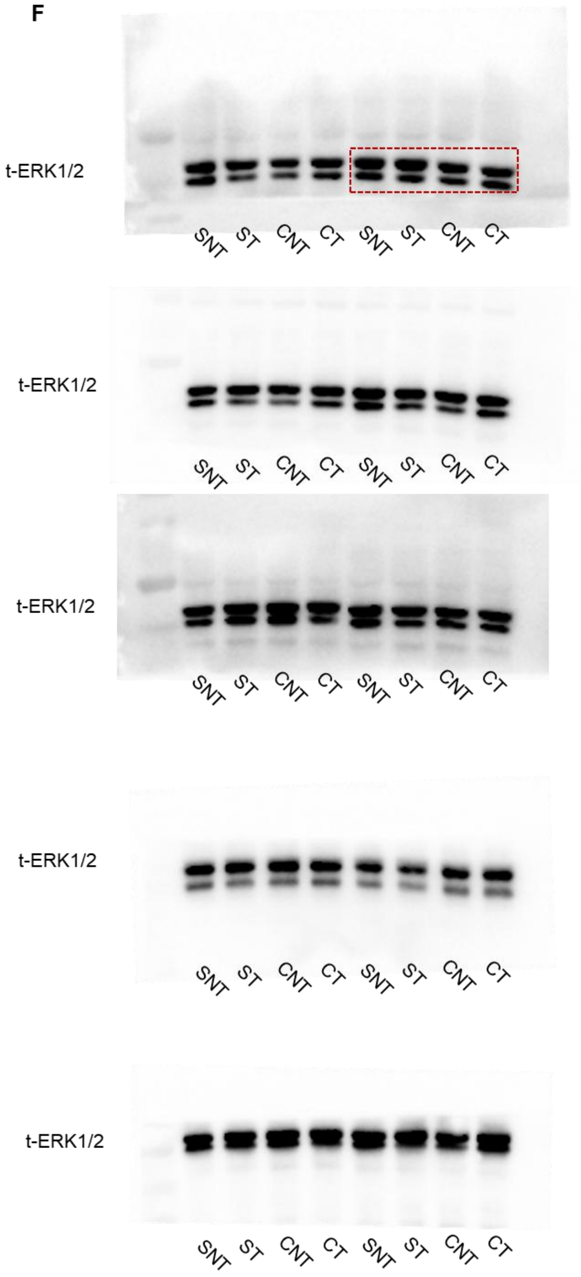
t-ERK1/2
SNT ST CNT CT SNT ST CNT CT
t-ERK1/2
SNT ST CNT CT SNT ST CNT CT
t-ERK1/2
SNT ST CNT CT SNT ST CNT CT
t-ERK1/2
SNT ST CNT CT SNT ST CNT CT
t-ERK1/2
SNT ST CNT CT SNT ST CNT CT

G

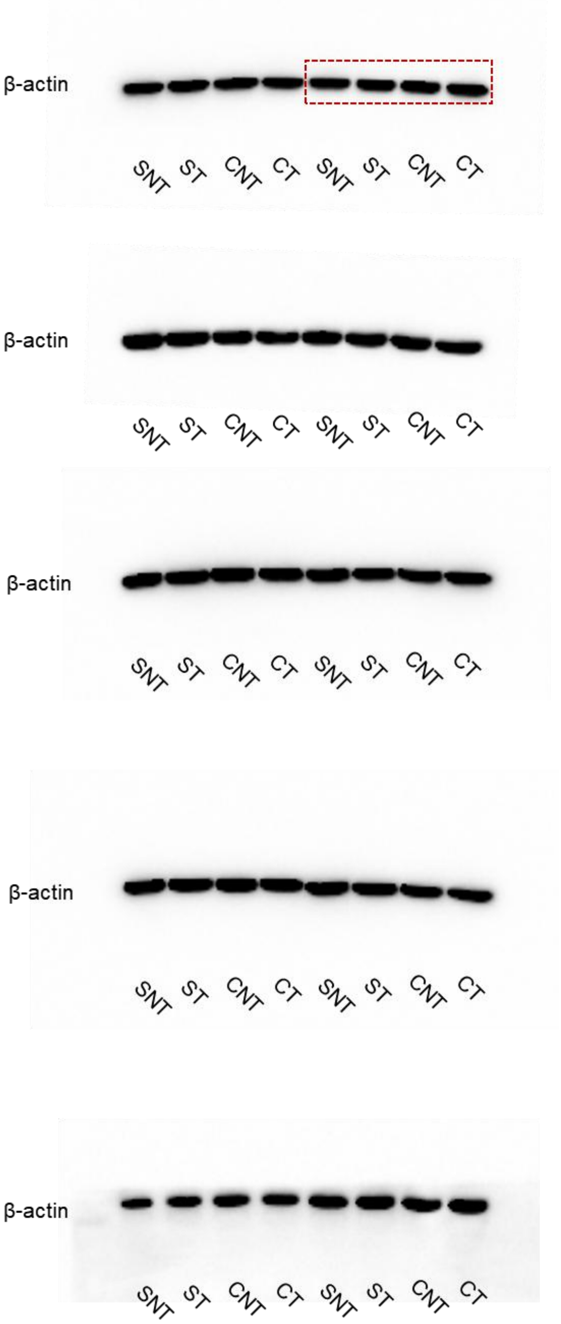

Supplementary Figure 1. Original full-length images of Western blots of phosphorylated AMPK (A), phosphorylated p70s6k (B), phosphorylated ERK1/2 (C), total AMPK (D), total p70s6k (E), total ERK1/2 (F), and β-actin (G) provided in Fig. 1E in the main text and all replicates. SNT, saline-no test; ST, saline-test; CNT, cocaine-no test; CT, cocaine-test.

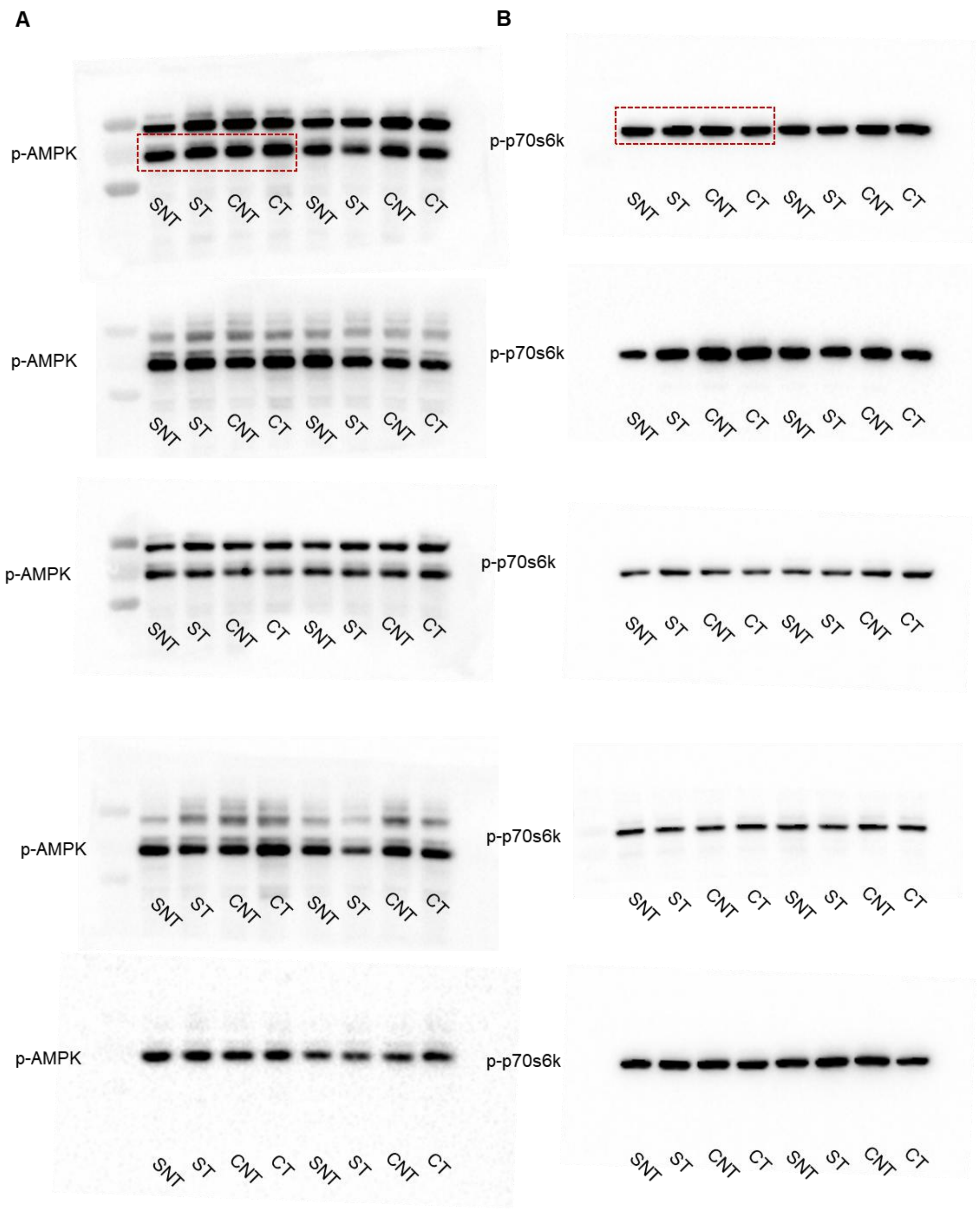
A
B
p-AMPK
SNT ST CNT CT SNT ST CNT CT
p-AMPK
SNT ST CNT CT SNT ST CNT CT
p-AMPK
SNT ST CNT CT SNT ST CNT CT
p-AMPK
SNT ST CNT CT SNT ST CNT CT
p-AMPK
SNT ST CNT CT SNT ST CNT CT
p-p70s6k
SNT ST CNT CT SNT ST CNT CT
p-p70s6k
SNT ST CNT CT SNT ST CNT CT
p-p70s6k
SNT ST CNT CT SNT ST CNT CT
p-p70s6k
SNT ST CNT CT SNT ST CNT CT
p-p70s6k
SNT ST CNT CT SNT ST CNT CT

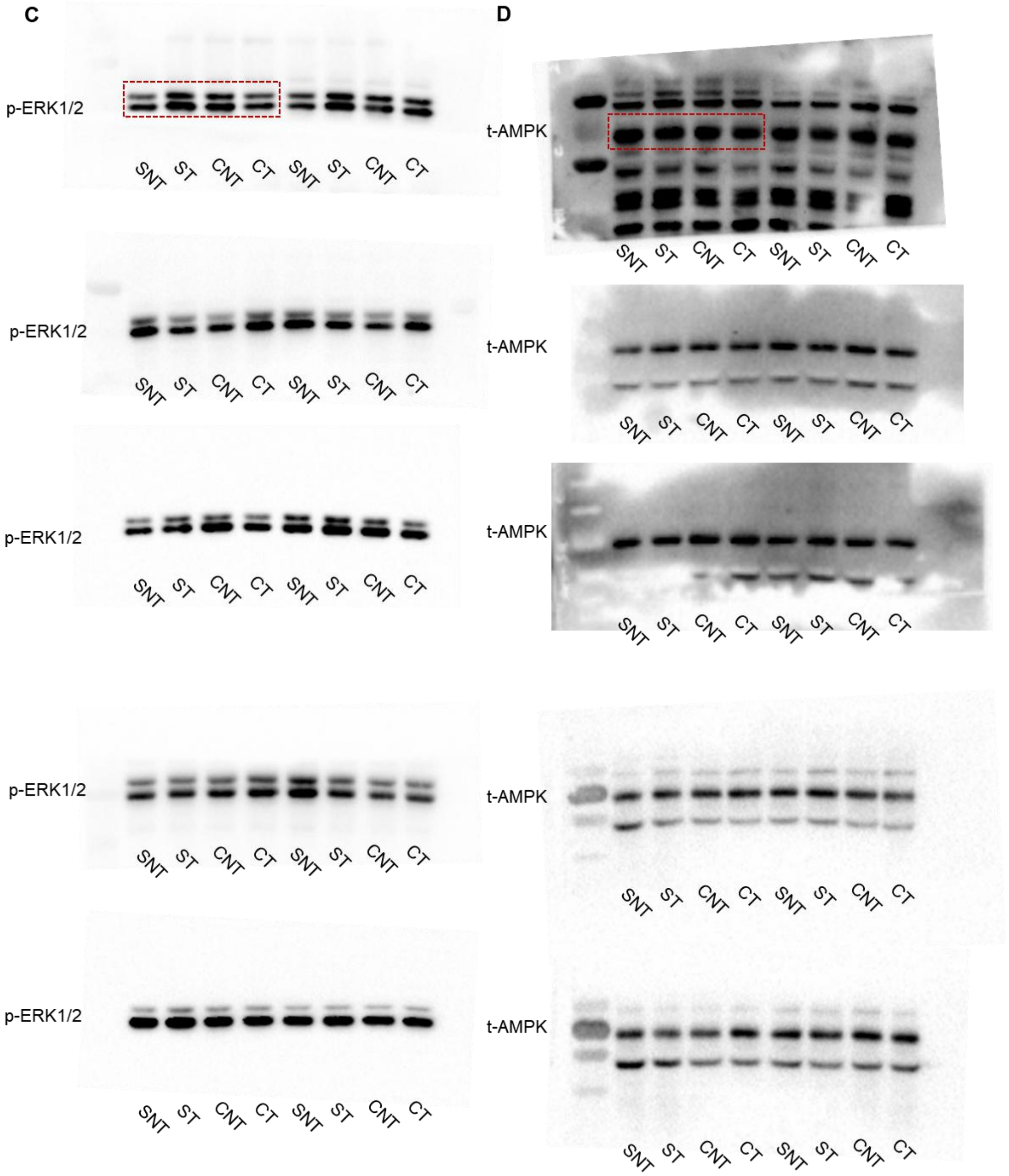
C
D
p-ERK1/2
SNT ST CNT CT SNT ST CNT CT
p-ERK1/2
SNT ST CNT CT SNT ST CNT CT
p-ERK1/2
SNT ST CNT CT SNT ST CNT CT
p-ERK1/2
SNT ST CNT CT SNT ST CNT CT
p-ERK1/2
SNT ST CNT CT SNT ST CNT CT
t-AMPK
SNT ST CNT CT SNT ST CNT CT
t-AMPK
SNT ST CNT CT SNT ST CNT CT
t-AMPK
SNT ST CNT CT SNT ST CNT CT
t-AMPK
SNT ST CNT CT SNT ST CNT CT
t-AMPK
SNT ST CNT CT SNT ST CNT CT

E

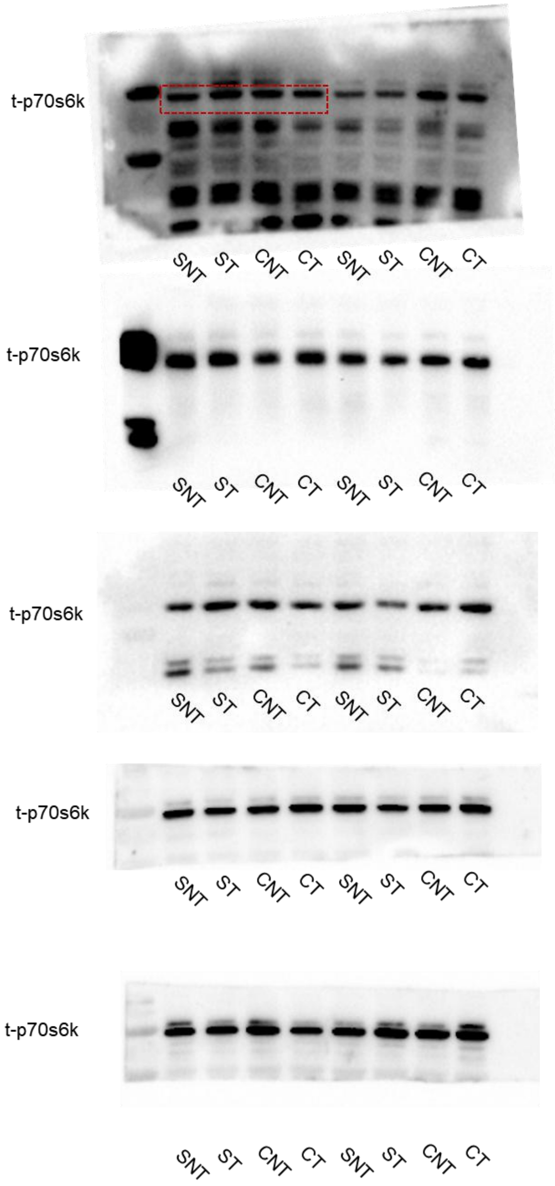
t-p70s6k
SNT ST CNT CT SNT ST CNT CT
t-p70s6k
SNT ST CNT CT SNT ST CNT CT
t-p70s6k
SNT ST CNT CT SNT ST CNT CT
t-p70s6k
SNT ST CNT CT SNT ST CNT CT
t-p70s6k
SNT ST CNT CT SNT ST CNT CT

F

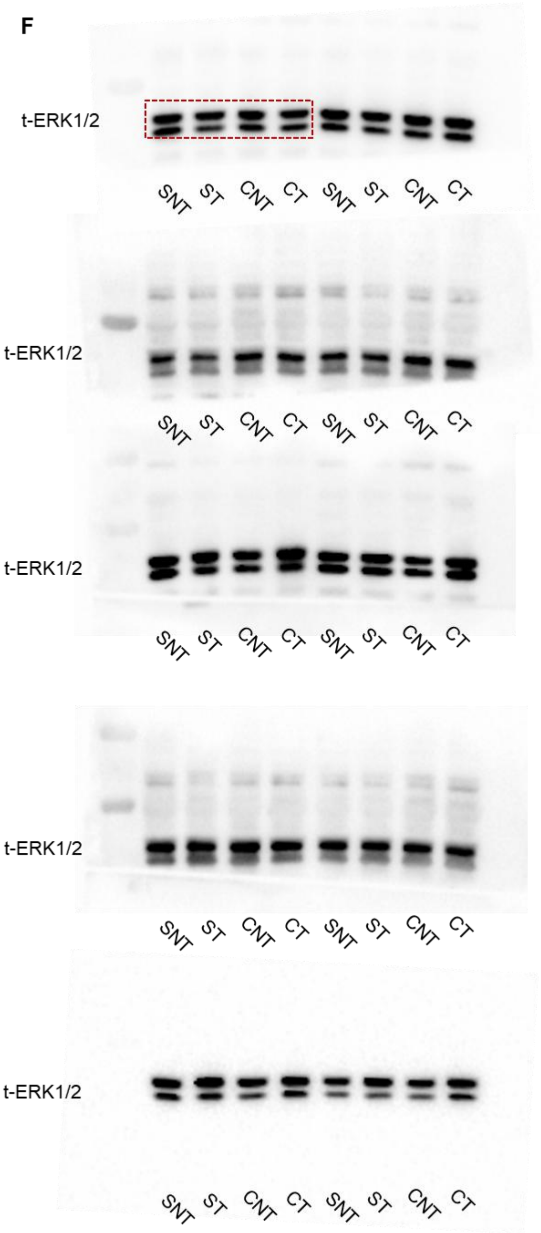
t-ERK1/2
SNT ST CNT CT SNT ST CNT CT
t-ERK1/2
SNT ST CNT CT SNT ST CNT CT
t-ERK1/2
SNT ST CNT CT SNT ST CNT CT
t-ERK1/2
SNT ST CNT CT SNT ST CNT CT
t-ERK1/2
SNT ST CNT CT SNT ST CNT CT

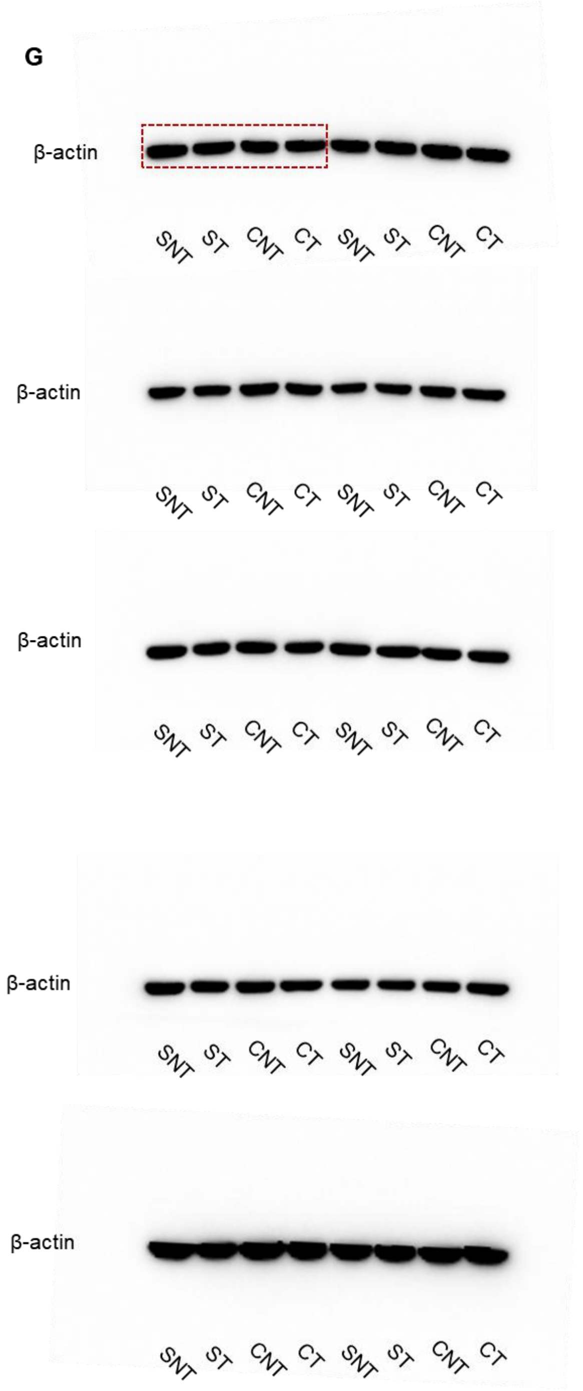

Supplementary Figure 2. Original full-length images of Western blots of phosphorylated AMPK (A), phosphorylated p70s6k (B), phosphorylated ERK1/2 (C), total AMPK (D), total p70s6k (E), total ERK1/2 (F), and β-actin (G) provided in Fig. 1F in the main text and all replicates. SNT, saline-no test; ST, saline-test; CNT, cocaine-no test; CT, cocaine-test.

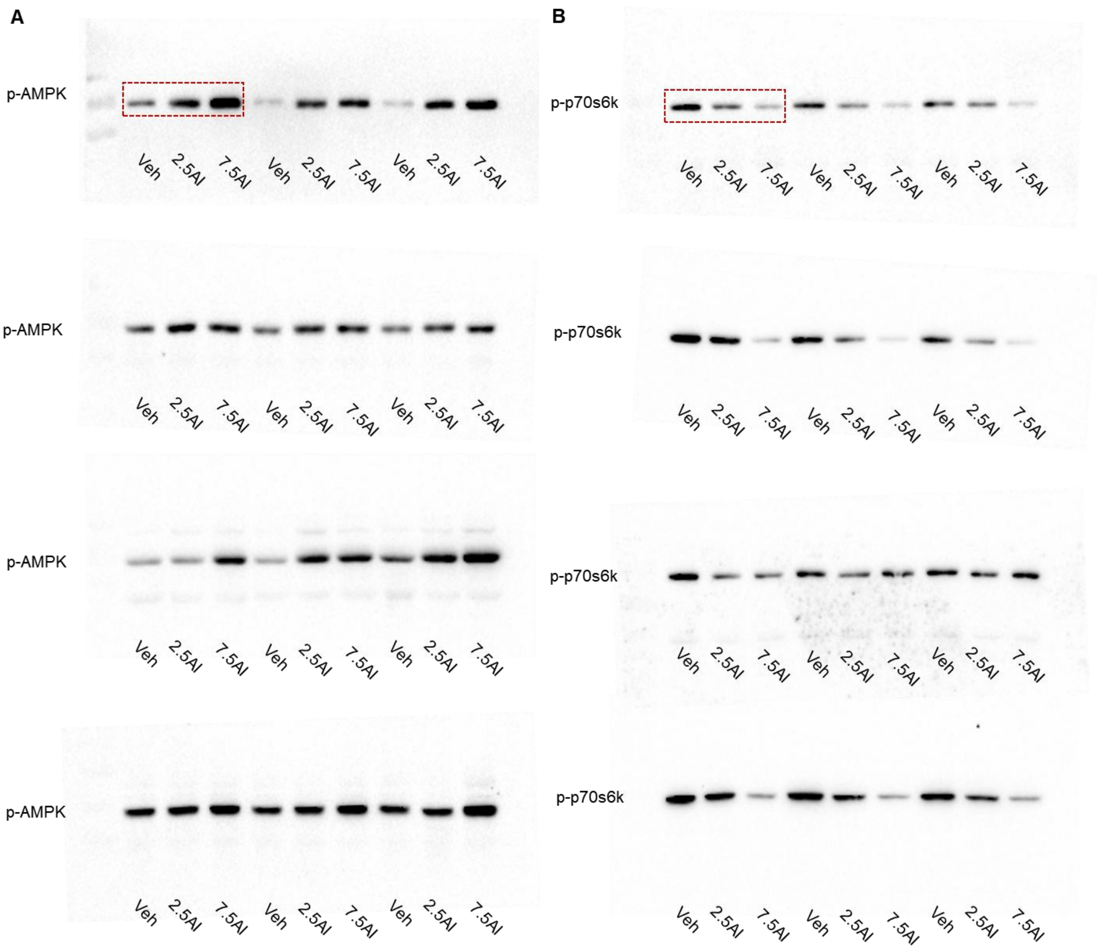
A
B
p-AMPK
p-p70s6k
Veh
2.5Al
7.5Al

C

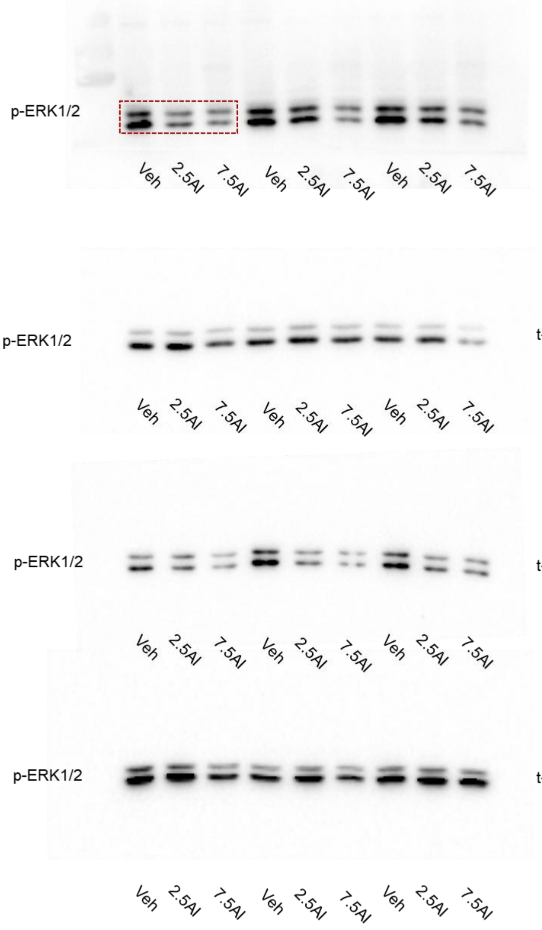
p-ERK1/2
Veh 2.5Al 7.5Al Veh 2.5Al 7.5Al Veh 2.5Al 7.5Al
p-ERK1/2
Veh 2.5Al 7.5Al Veh 2.5Al 7.5Al Veh 2.5Al 7.5Al
p-ERK1/2
Veh 2.5Al 7.5Al Veh 2.5Al 7.5Al Veh 2.5Al 7.5Al
p-ERK1/2
Veh 2.5Al 7.5Al Veh 2.5Al 7.5Al Veh 2.5Al 7.5Al

D

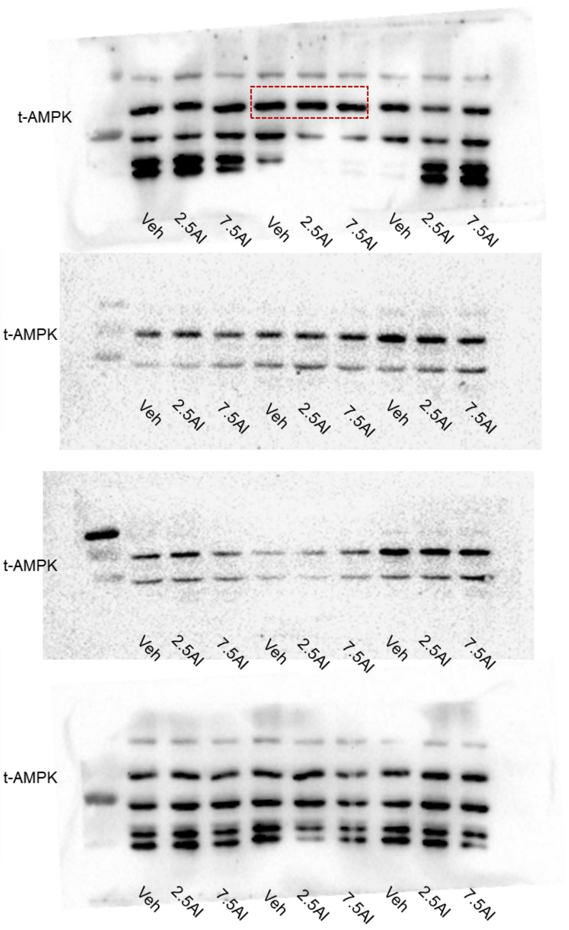
t-AMPK
Veh 2.5Al 7.5Al Veh 2.5Al 7.5Al Veh 2.5Al 7.5Al
t-AMPK
Veh 2.5Al 7.5Al Veh 2.5Al 7.5Al Veh 2.5Al 7.5Al
t-AMPK
Veh 2.5Al 7.5Al Veh 2.5Al 7.5Al Veh 2.5Al 7.5Al
t-AMPK
Veh 2.5Al 7.5Al Veh 2.5Al 7.5Al Veh 2.5Al 7.5Al

E

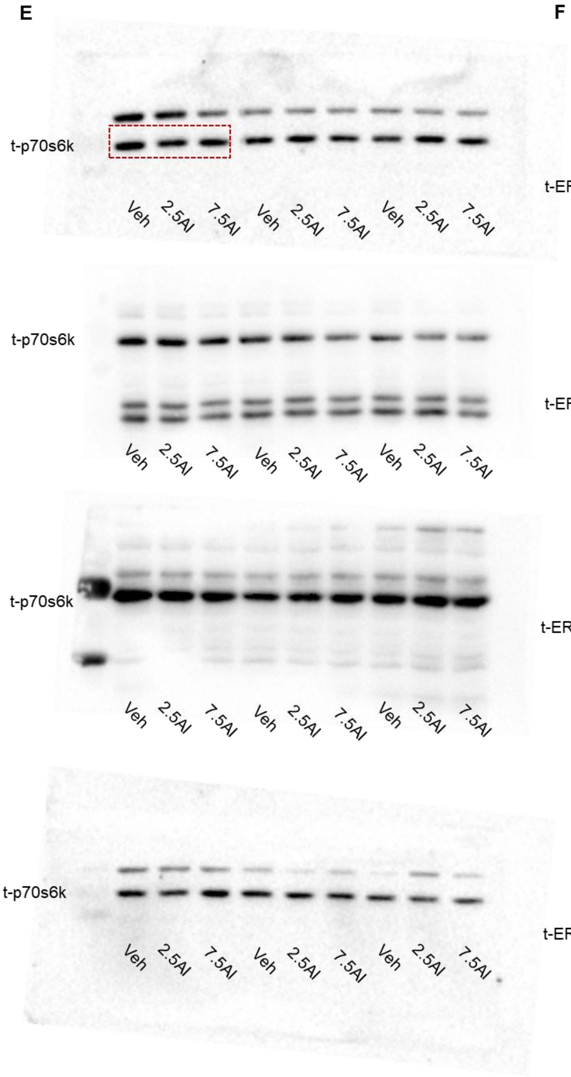
t-p70s6k
Veh 2.5AI 7.5AI Veh 2.5AI 7.5AI Veh 2.5AI 7.5AI
t-p70s6k
Veh 2.5AI 7.5AI Veh 2.5AI 7.5AI Veh 2.5AI 7.5AI
t-p70s6k
Veh 2.5AI 7.5AI Veh 2.5AI 7.5AI Veh 2.5AI 7.5AI
t-p70s6k
Veh 2.5AI 7.5AI Veh 2.5AI 7.5AI Veh 2.5AI 7.5AI

F

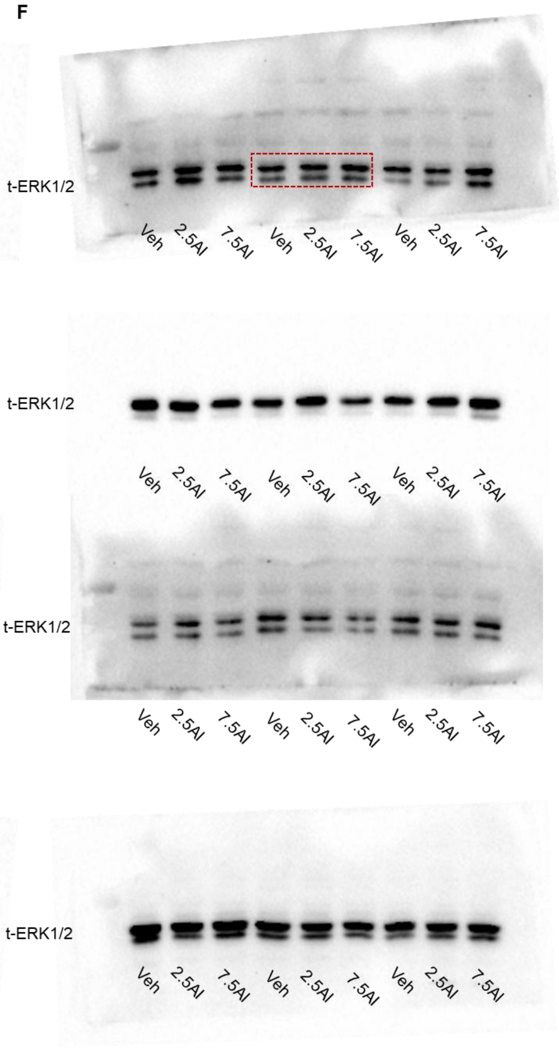
t-ERK1/2
Veh 2.5AI 7.5AI Veh 2.5AI 7.5AI Veh 2.5AI 7.5AI
t-ERK1/2
Veh 2.5AI 7.5AI Veh 2.5AI 7.5AI Veh 2.5AI 7.5AI
t-ERK1/2
Veh 2.5AI 7.5AI Veh 2.5AI 7.5AI Veh 2.5AI 7.5AI
t-ERK1/2
Veh 2.5AI 7.5AI Veh 2.5AI 7.5AI Veh 2.5AI 7.5AI

G

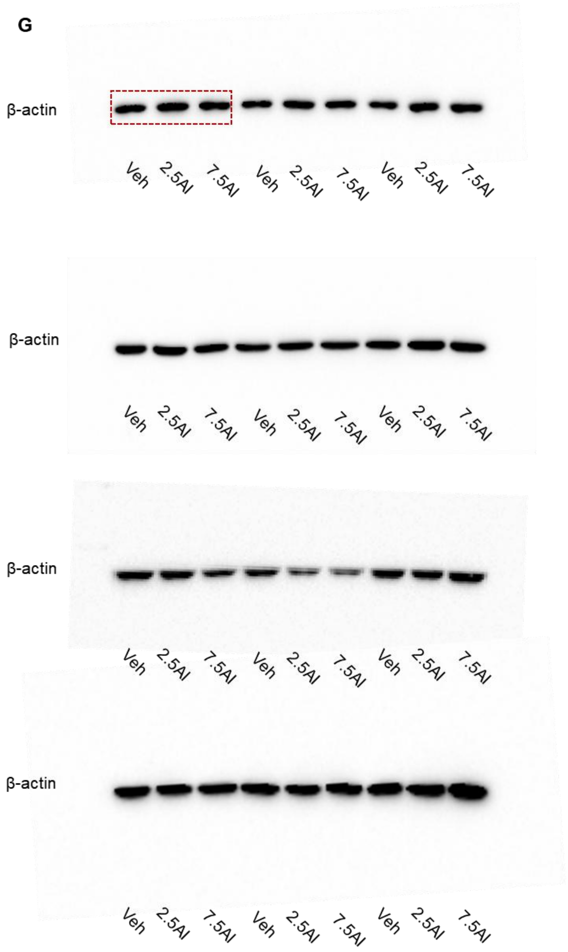

Supplementary Figure 3. Original full-length images of Western blots of phosphorylated AMPK (A), phosphorylated p70s6k (B), phosphorylated ERK1/2 (C), total AMPK (D), total p70s6k (E), total ERK1/2 (F), and β-actin (G) provided in Fig. 2E in the main text and all replicates. Veh, vehicle; 2.5AI, 2.5μg AICAR; 7.5AI, 7.5μg AICAR.

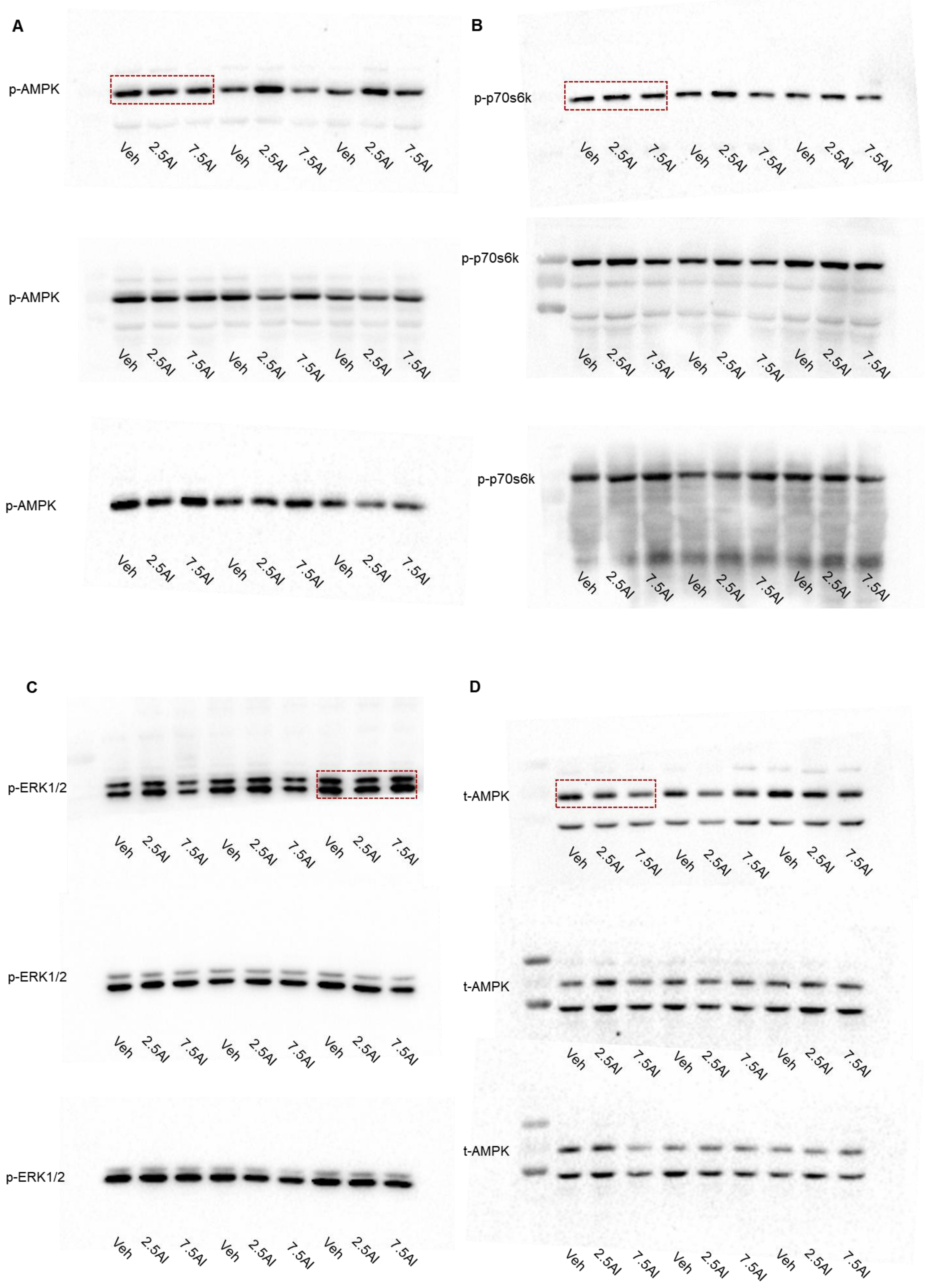
A
p-AMPK
Veh 2.5AI 7.5AI Veh 2.5AI 7.5AI Veh 2.5AI 7.5AI
p-AMPK
Veh 2.5AI 7.5AI Veh 2.5AI 7.5AI Veh 2.5AI 7.5AI
p-AMPK
Veh 2.5AI 7.5AI Veh 2.5AI 7.5AI Veh 2.5AI 7.5AI
B
p-p70s6k
Veh 2.5AI 7.5AI Veh 2.5AI 7.5AI Veh 2.5AI 7.5AI
p-p70s6k
Veh 2.5AI 7.5AI Veh 2.5AI 7.5AI Veh 2.5AI 7.5AI
p-p70s6k
Veh 2.5AI 7.5AI Veh 2.5AI 7.5AI Veh 2.5AI 7.5AI
C
p-ERK1/2
Veh 2.5AI 7.5AI Veh 2.5AI 7.5AI Veh 2.5AI 7.5AI
p-ERK1/2
Veh 2.5AI 7.5AI Veh 2.5AI 7.5AI Veh 2.5AI 7.5AI
p-ERK1/2
Veh 2.5AI 7.5AI Veh 2.5AI 7.5AI Veh 2.5AI 7.5AI
D
t-AMPK
Veh 2.5AI 7.5AI Veh 2.5AI 7.5AI Veh 2.5AI 7.5AI
t-AMPK
Veh 2.5AI 7.5AI Veh 2.5AI 7.5AI Veh 2.5AI 7.5AI
t-AMPK
Veh 2.5AI 7.5AI Veh 2.5AI 7.5AI Veh 2.5AI 7.5AI

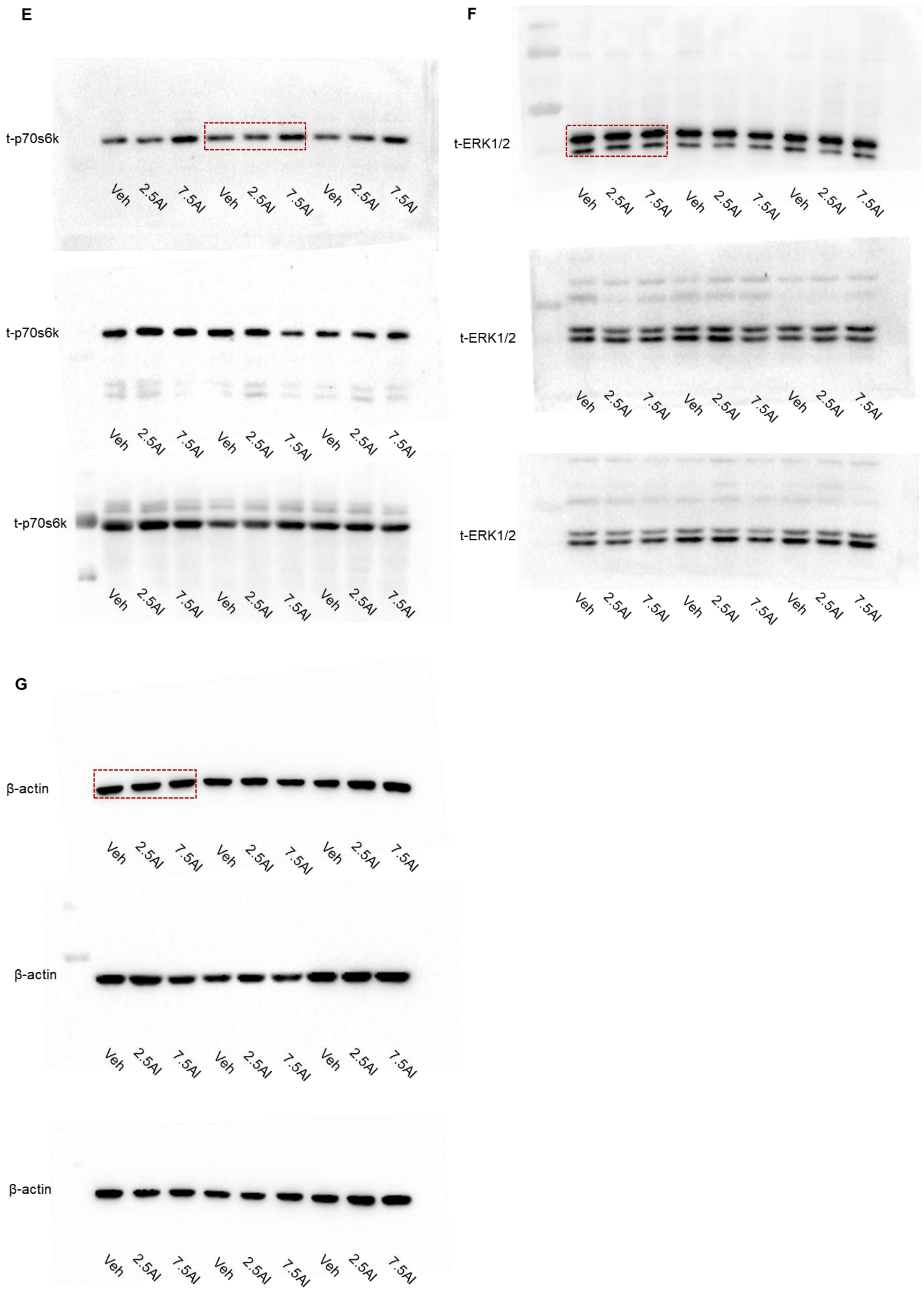

Supplementary Figure 4. Original full-length images of Western blots of phosphorylated AMPK (A), phosphorylated p70s6k (B), phosphorylated ERK1/2 (C), total AMPK (D), total p70s6k (E), total ERK1/2 (F), and β-actin (G) provided in Fig. 2F in the main text and all replicates. Veh, vehicle; 2.5AI, 2.5μg AICAR; 7.5AI, 7.5μg AICAR.

A

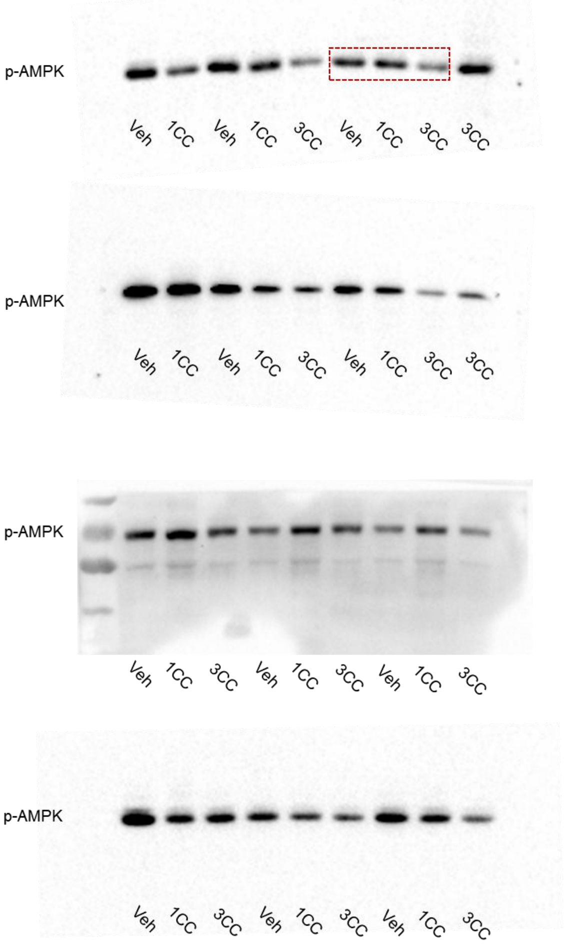

p-AMPK
Veh 1CC Veh 1CC 3CC Veh 1CC 3CC 3CC
p-AMPK
Veh 1CC Veh 1CC 3CC Veh 1CC 3CC 3CC
p-AMPK
Veh 1CC 3CC Veh 1CC 3CC Veh 1CC 3CC
p-AMPK
Veh 1CC 3CC Veh 1CC 3CC Veh 1CC 3CC

B

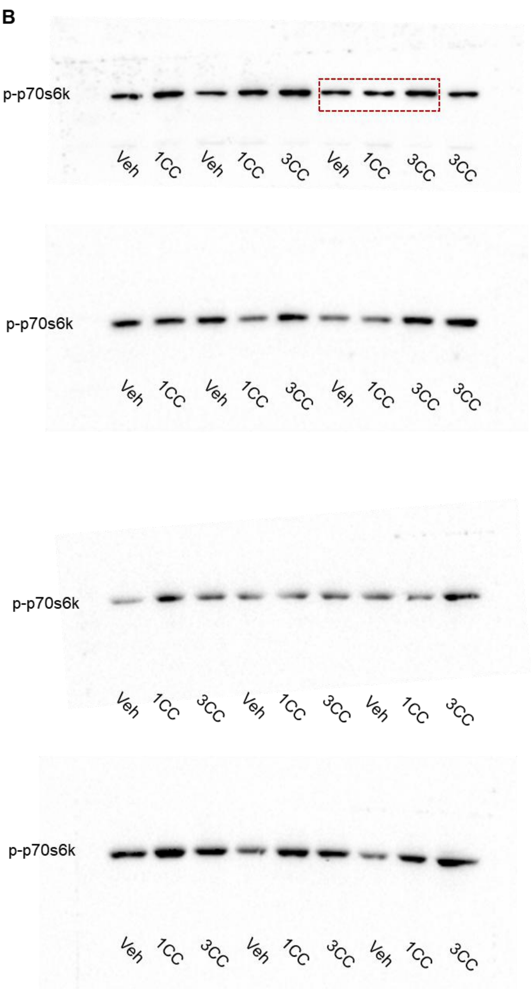

p-p70s6k
Veh 1CC Veh 1CC 3CC Veh 1CC 3CC 3CC
p-p70s6k
Veh 1CC Veh 1CC 3CC Veh 1CC 3CC 3CC
p-p70s6k
Veh 1CC 3CC Veh 1CC 3CC Veh 1CC 3CC
p-p70s6k
Veh 1CC 3CC Veh 1CC 3CC Veh 1CC 3CC

C

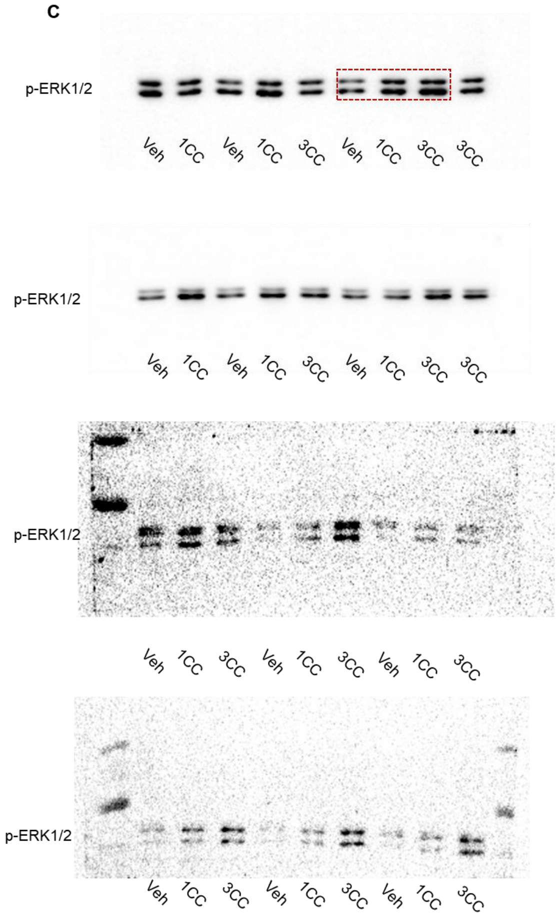
p-ERK1/2
Veh 1CC Veh 1CC 3CC Veh 1CC 3CC 3CC
p-ERK1/2
Veh 1CC Veh 1CC 3CC Veh 1CC 3CC 3CC
p-ERK1/2
Veh 1CC 3CC Veh 1CC 3CC Veh 1CC 3CC
p-ERK1/2
Veh 1CC 3CC Veh 1CC 3CC Veh 1CC 3CC

D

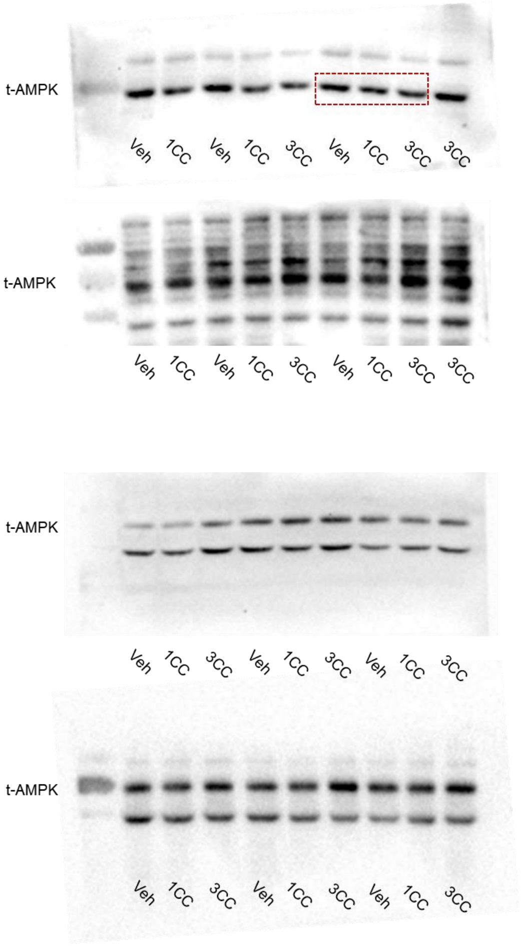
t-AMPK
Veh 1CC Veh 1CC 3CC Veh 1CC 3CC 3CC
t-AMPK
Veh 1CC Veh 1CC 3CC Veh 1CC 3CC 3CC
t-AMPK
Veh 1CC 3CC Veh 1CC 3CC Veh 1CC 3CC
t-AMPK
Veh 1CC 3CC Veh 1CC 3CC Veh 1CC 3CC

E

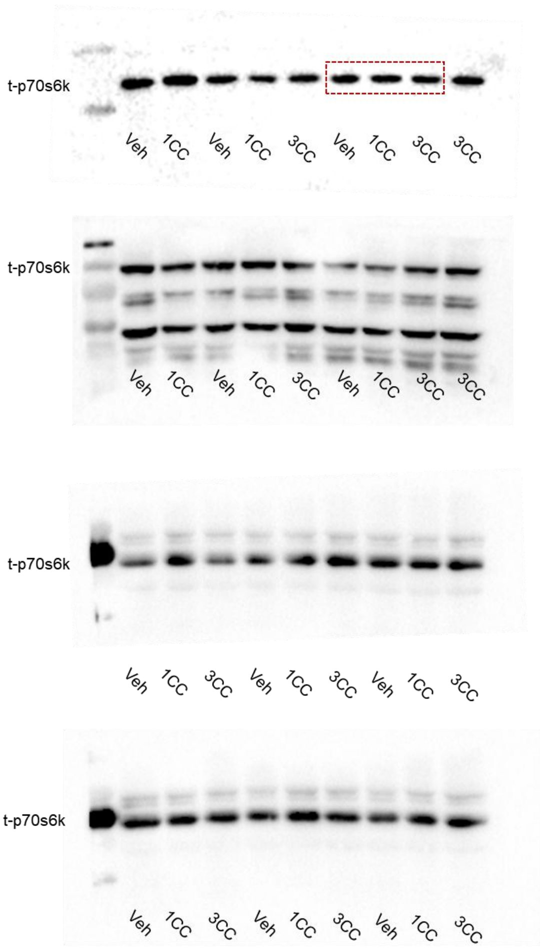
t-p70s6k
Veh 1CC Veh 1CC 3CC Veh 1CC 3CC 3CC
t-p70s6k
Veh 1CC Veh 1CC 3CC Veh 1CC 3CC 3CC
t-p70s6k
Veh 1CC 3CC Veh 1CC 3CC Veh 1CC 3CC
t-p70s6k
Veh 1CC 3CC Veh 1CC 3CC Veh 1CC 3CC

F

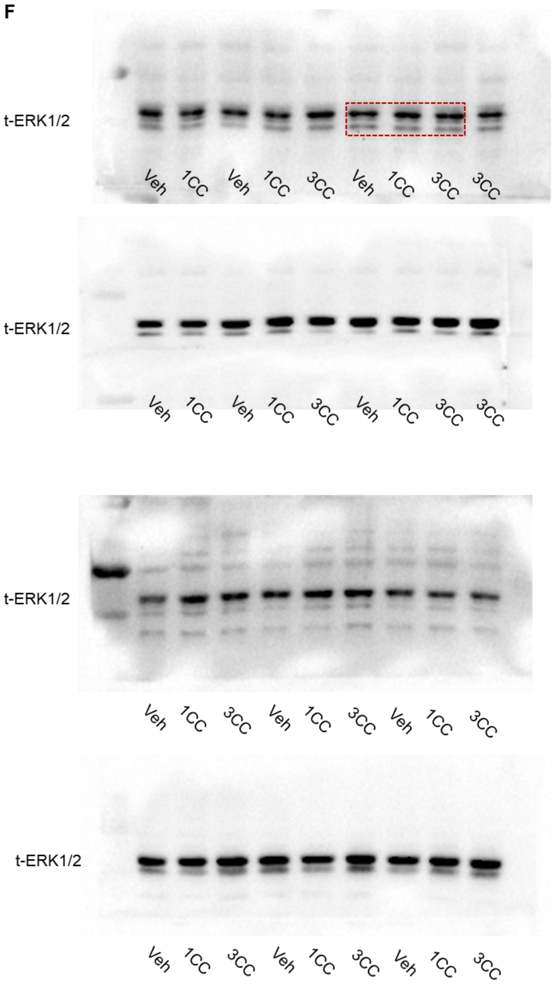
t-ERK1/2
Veh 1CC Veh 1CC 3CC Veh 1CC 3CC 3CC
t-ERK1/2
Veh 1CC Veh 1CC 3CC Veh 1CC 3CC 3CC
t-ERK1/2
Veh 1CC 3CC Veh 1CC 3CC Veh 1CC 3CC
t-ERK1/2
Veh 1CC 3CC Veh 1CC 3CC Veh 1CC 3CC

G

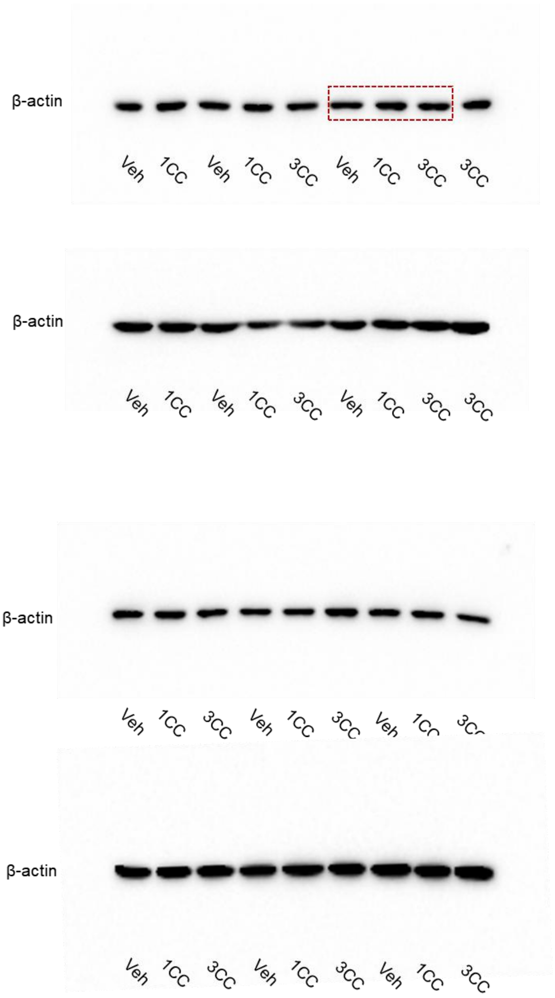

Supplementary Figure 5. Original full-length images of Western blots of phosphorylated AMPK (A), phosphorylated p70s6k (B), phosphorylated ERK1/2 (C), total AMPK (D), total p70s6k (E), total ERK1/2 (F), and β-actin (G) provided in Fig. 3E in the main text and all replicates. Veh, vehicle; 1CC, 1µg comp C; 3CC, 3µg comp C.

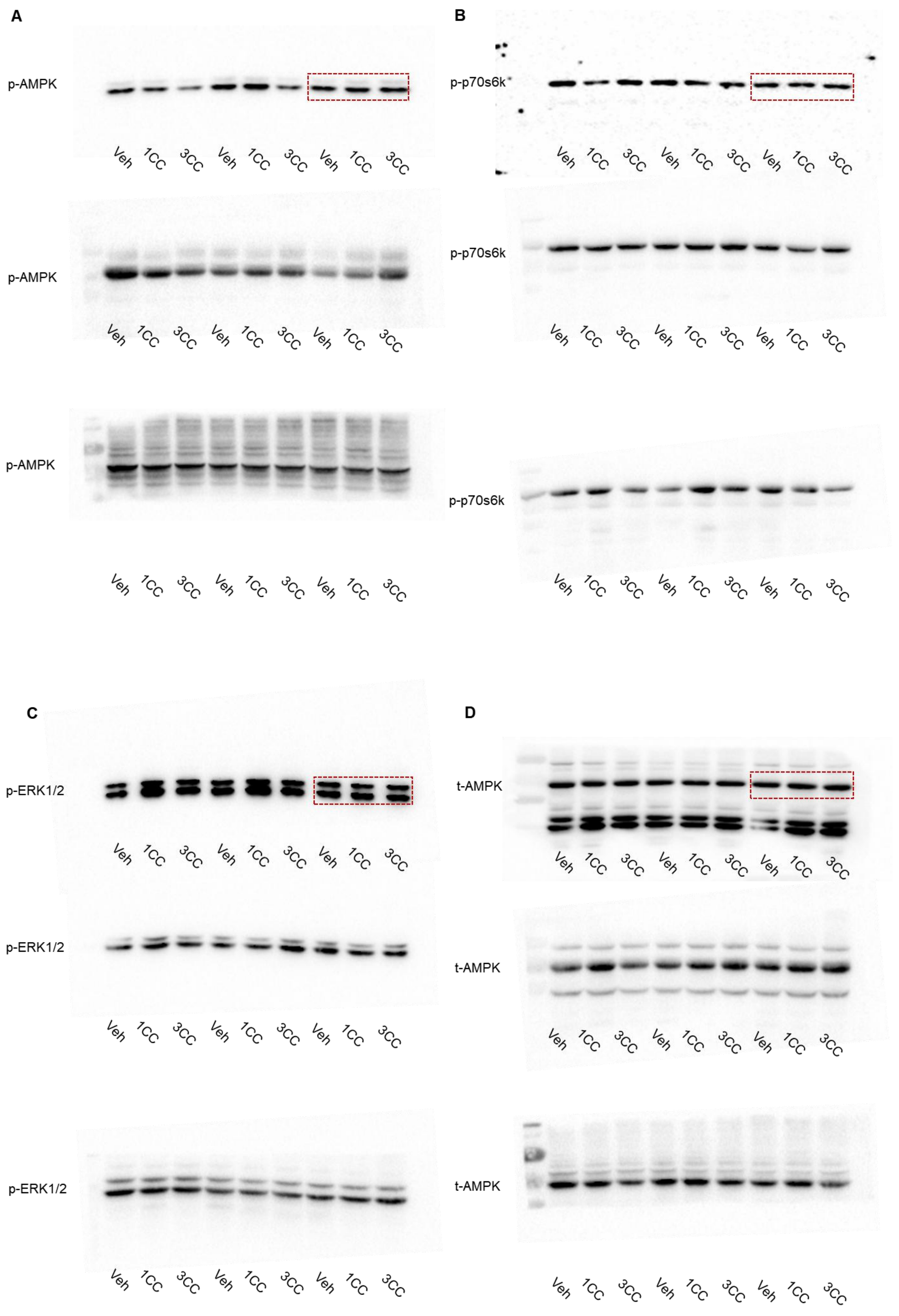
A
p-AMPK
Veh 1CC 3CC Veh 1CC 3CC Veh 1CC 3CC
p-AMPK
Veh 1CC 3CC Veh 1CC 3CC Veh 1CC 3CC
p-AMPK
Veh 1CC 3CC Veh 1CC 3CC Veh 1CC 3CC
B
p-p70s6k
Veh 1CC 3CC Veh 1CC 3CC Veh 1CC 3CC
p-p70s6k
Veh 1CC 3CC Veh 1CC 3CC Veh 1CC 3CC
p-p70s6k
Veh 1CC 3CC Veh 1CC 3CC Veh 1CC 3CC
C
p-ERK1/2
Veh 1CC 3CC Veh 1CC 3CC Veh 1CC 3CC
p-ERK1/2
Veh 1CC 3CC Veh 1CC 3CC Veh 1CC 3CC
p-ERK1/2
Veh 1CC 3CC Veh 1CC 3CC Veh 1CC 3CC
D
t-AMPK
Veh 1CC 3CC Veh 1CC 3CC Veh 1CC 3CC
t-AMPK
Veh 1CC 3CC Veh 1CC 3CC Veh 1CC 3CC
t-AMPK
Veh 1CC 3CC Veh 1CC 3CC Veh 1CC 3CC

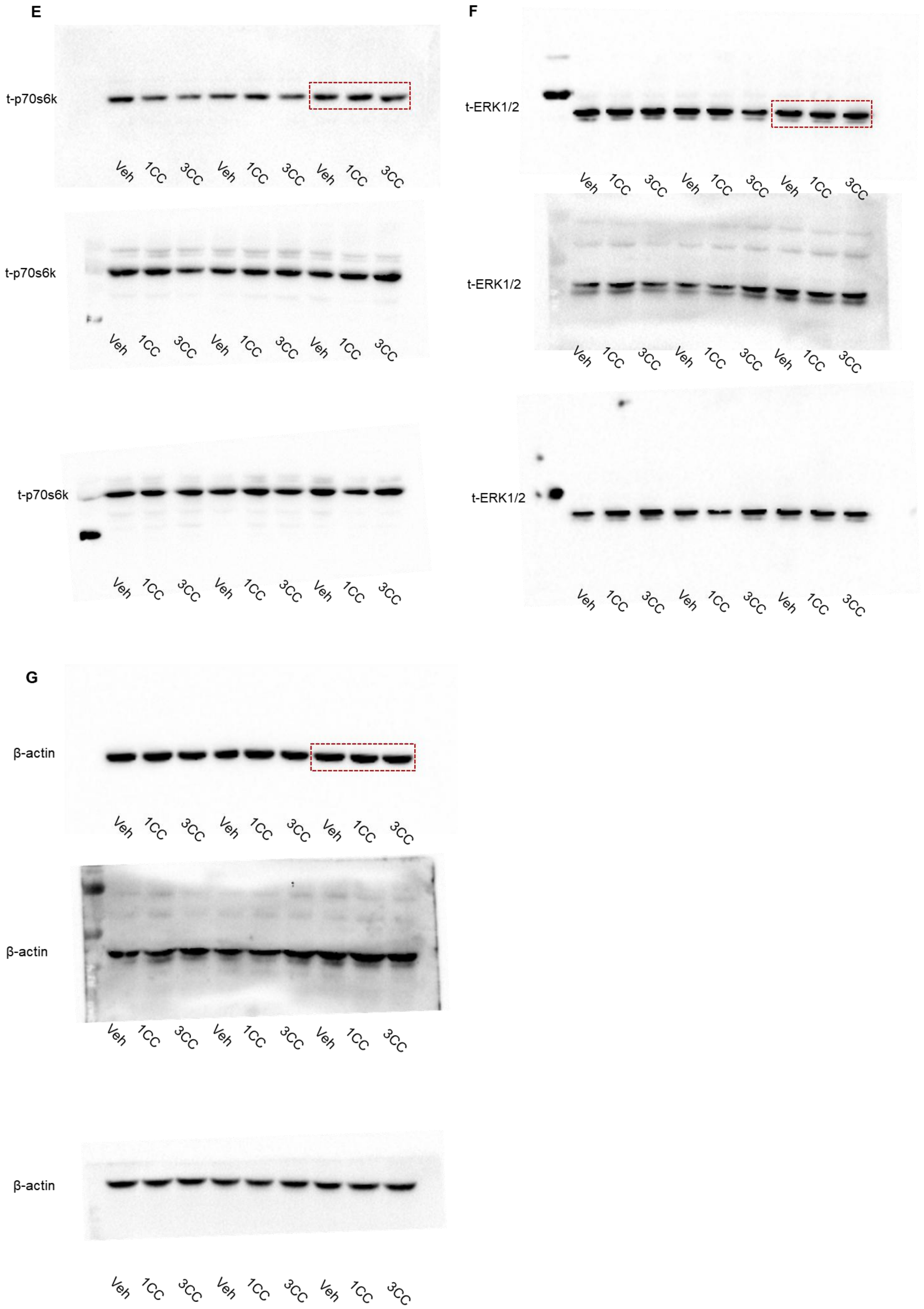

Supplementary Figure 6. Original full-length images of Western blots of phosphorylated AMPK (A), phosphorylated p70s6k (B), phosphorylated ERK1/2 (C), total AMPK (D), total p70s6k (E), total ERK1/2 (F), and β-actin (G) provided in Fig. 3F in the main text and all replicates. Veh, vehicle; 1CC, 1μg comp C; 3CC, 3μg comp C.

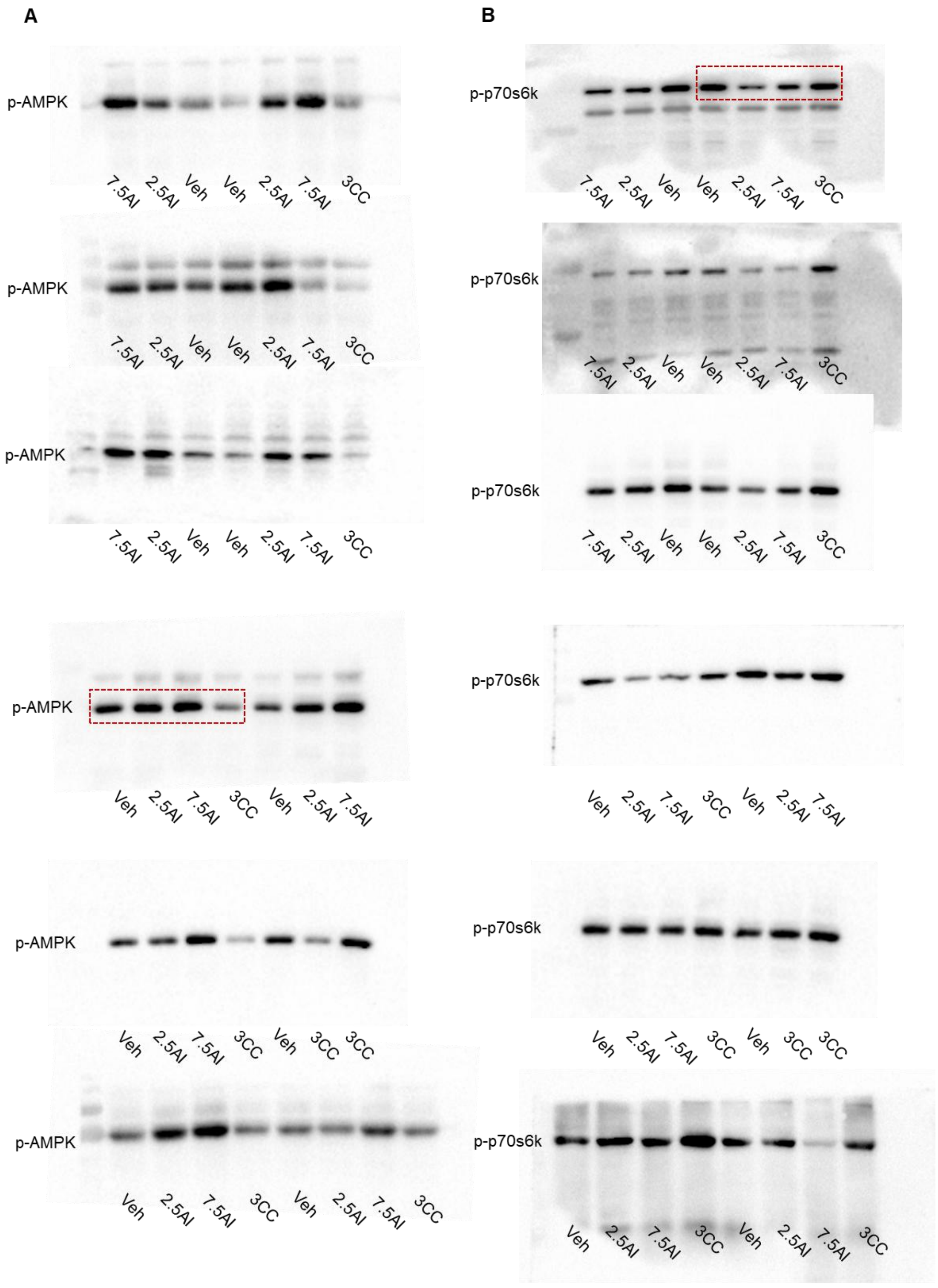

A
p-AMPK
7.5Al 2.5Al Veh Veh 2.5Al 7.5Al 3CC
p-AMPK
7.5Al 2.5Al Veh Veh 2.5Al 7.5Al 3CC
p-AMPK
7.5Al 2.5Al Veh Veh 2.5Al 7.5Al 3CC
p-AMPK
Veh 2.5Al 7.5Al 3CC Veh 2.5Al 7.5Al
p-AMPK
Veh 2.5Al 7.5Al 3CC Veh 3CC 3CC
p-AMPK
Veh 2.5Al 7.5Al 3CC Veh 2.5Al 7.5Al 3CC
B
p-p70s6k
7.5Al 2.5Al Veh Veh 2.5Al 7.5Al 3CC
p-p70s6k
7.5Al 2.5Al Veh Veh 2.5Al 7.5Al 3CC
p-p70s6k
7.5Al 2.5Al Veh Veh 2.5Al 7.5Al 3CC
p-p70s6k
Veh 2.5Al 7.5Al 3CC Veh 2.5Al 7.5Al
p-p70s6k
Veh 2.5Al 7.5Al 3CC Veh 3CC 3CC
p-p70s6k
Veh 2.5Al 7.5Al 3CC Veh 2.5Al 7.5Al 3CC

C

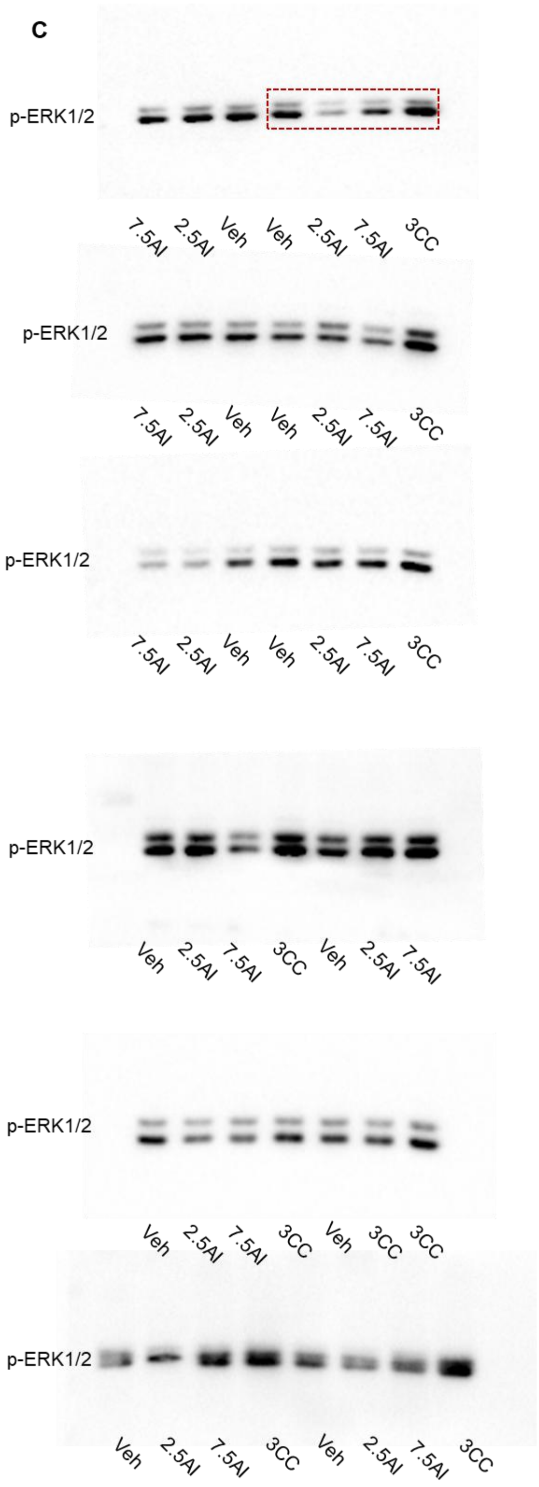
p-ERK1/2
7.5AI 2.5AI Veh Veh 2.5AI 7.5AI 3CC
p-ERK1/2
7.5AI 2.5AI Veh Veh 2.5AI 7.5AI 3CC
p-ERK1/2
7.5AI 2.5AI Veh Veh 2.5AI 7.5AI 3CC
p-ERK1/2
Veh 2.5AI 7.5AI 3CC Veh 2.5AI 7.5AI
p-ERK1/2
Veh 2.5AI 7.5AI 3CC Veh 3CC 3CC
p-ERK1/2
Veh 2.5AI 7.5AI 3CC Veh 2.5AI 7.5AI 3CC

D

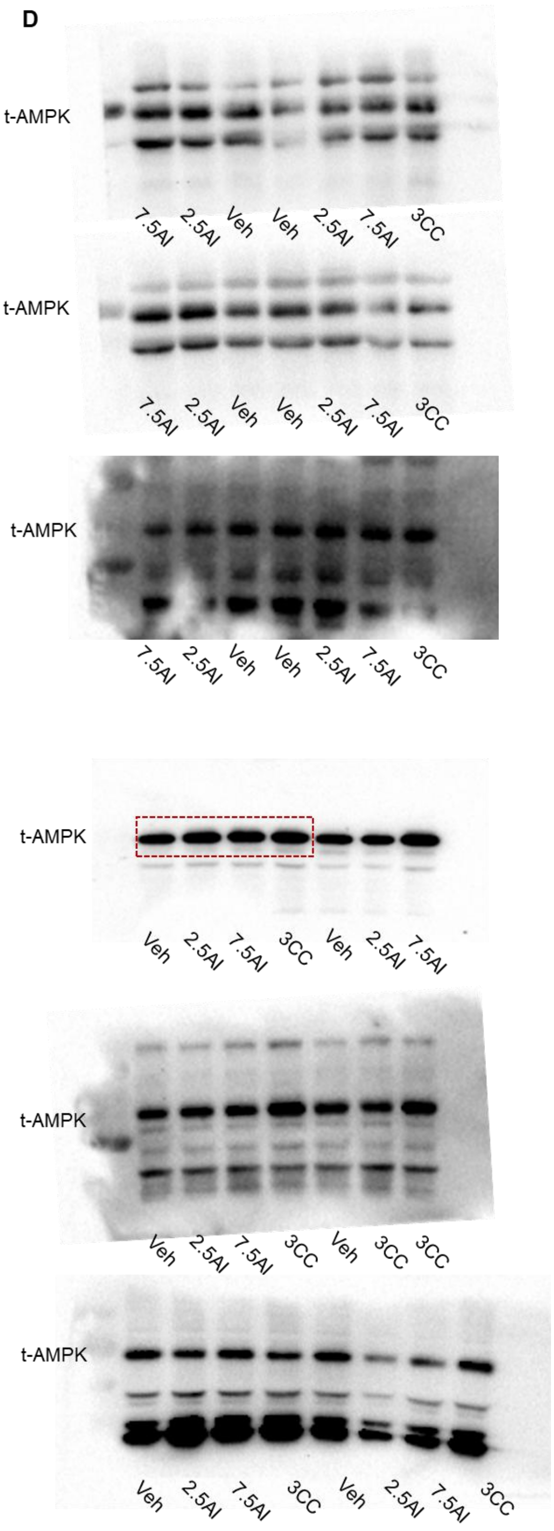
t-AMPK
7.5AI 2.5AI Veh Veh 2.5AI 7.5AI 3CC
t-AMPK
7.5AI 2.5AI Veh Veh 2.5AI 7.5AI 3CC
t-AMPK
7.5AI 2.5AI Veh Veh 2.5AI 7.5AI 3CC
t-AMPK
Veh 2.5AI 7.5AI 3CC Veh 2.5AI 7.5AI
t-AMPK
Veh 2.5AI 7.5AI 3CC Veh 3CC 3CC
t-AMPK
Veh 2.5AI 7.5AI 3CC Veh 2.5AI 7.5AI 3CC

E

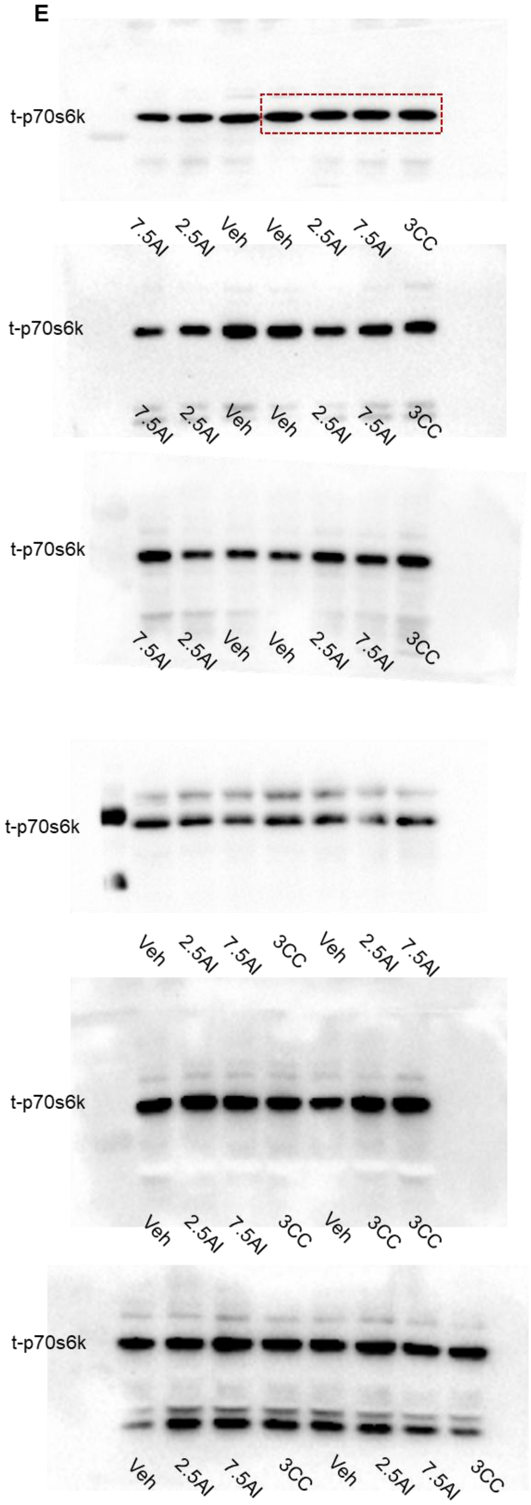
t-p70s6k
7.5AI 2.5AI Veh Veh 2.5AI 7.5AI 3CC
t-p70s6k
7.5AI 2.5AI Veh Veh 2.5AI 7.5AI 3CC
t-p70s6k
7.5AI 2.5AI Veh Veh 2.5AI 7.5AI 3CC
t-p70s6k
Veh 2.5AI 7.5AI 3CC Veh 2.5AI 7.5AI
t-p70s6k
Veh 2.5AI 7.5AI 3CC Veh 3CC 3CC
t-p70s6k
Veh 2.5AI 7.5AI 3CC Veh 2.5AI 7.5AI 3CC

F

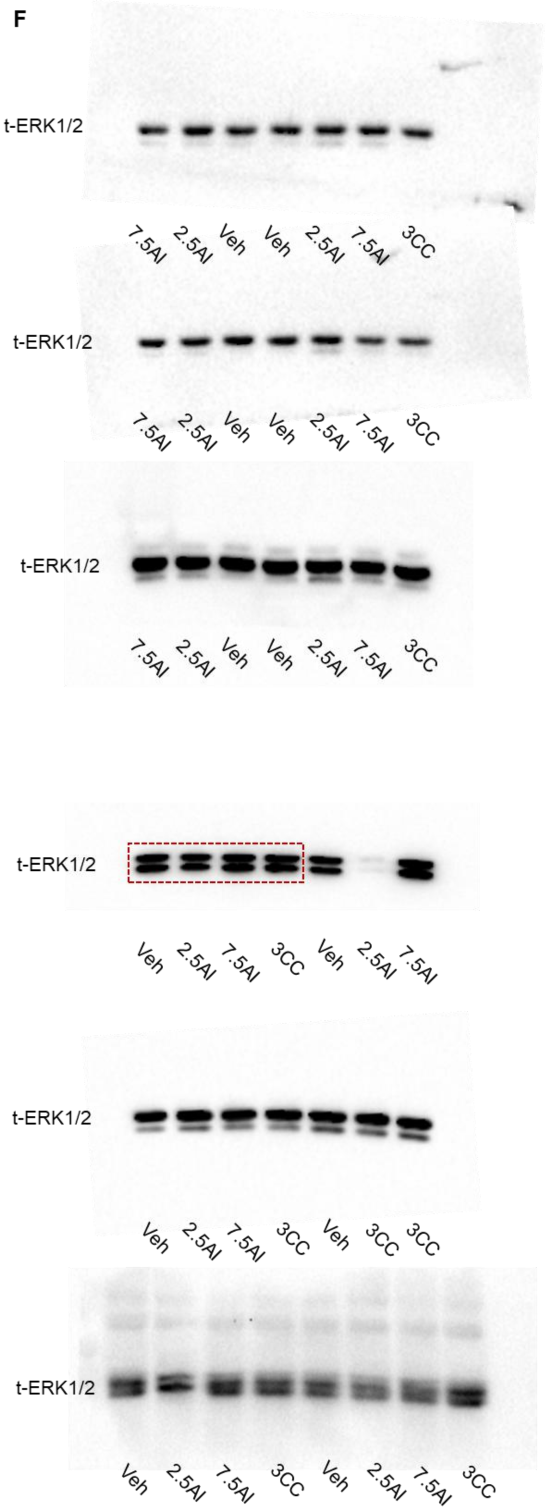
t-ERK1/2
7.5AI 2.5AI Veh Veh 2.5AI 7.5AI 3CC
t-ERK1/2
7.5AI 2.5AI Veh Veh 2.5AI 7.5AI 3CC
t-ERK1/2
7.5AI 2.5AI Veh Veh 2.5AI 7.5AI 3CC
t-ERK1/2
Veh 2.5AI 7.5AI 3CC Veh 2.5AI 7.5AI
t-ERK1/2
Veh 2.5AI 7.5AI 3CC Veh 3CC 3CC
t-ERK1/2
Veh 2.5AI 7.5AI 3CC Veh 2.5AI 7.5AI 3CC

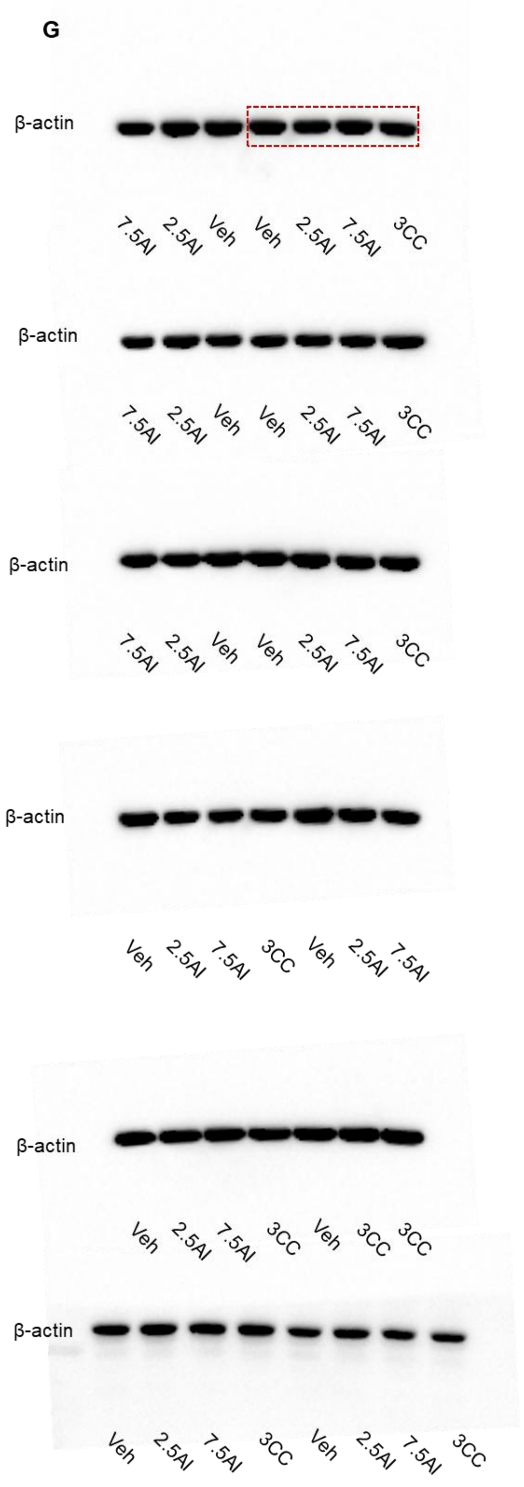

Supplementary Figure 7. Original full-length images of Western blots of phosphorylated AMPK (A), phosphorylated p70s6k (B), phosphorylated ERK1/2 (C), total AMPK (D), total p70s6k (E), total ERK1/2 (F), and β-actin (G) provided in Fig. 4E in the main text and all replicates. Veh, vehicle; 2.5AI, 2.5μg AICAR; 7.5AI, 7.5μg AICAR; 3CC, 3μg comp C.

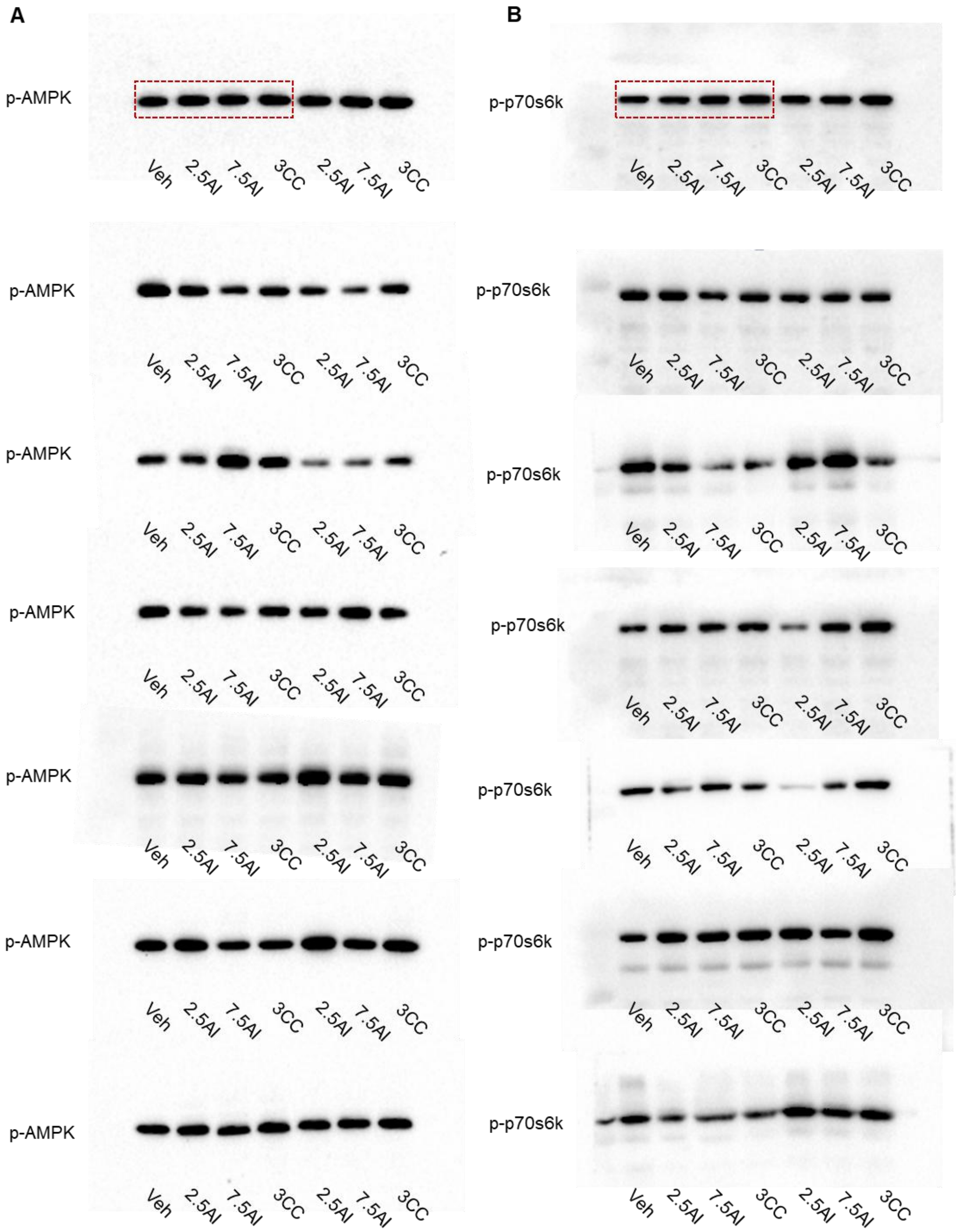
A
p-AMPK
Veh 2.5AI 7.5AI 3CC 2.5AI 7.5AI 3CC
p-AMPK
Veh 2.5AI 7.5AI 3CC 2.5AI 7.5AI 3CC
p-AMPK
Veh 2.5AI 7.5AI 3CC 2.5AI 7.5AI 3CC
p-AMPK
Veh 2.5AI 7.5AI 3CC 2.5AI 7.5AI 3CC
p-AMPK
Veh 2.5AI 7.5AI 3CC 2.5AI 7.5AI 3CC
p-AMPK
Veh 2.5AI 7.5AI 3CC 2.5AI 7.5AI 3CC
p-AMPK
Veh 2.5AI 7.5AI 3CC 2.5AI 7.5AI 3CC
B
p-p70s6k
Veh 2.5AI 7.5AI 3CC 2.5AI 7.5AI 3CC
p-p70s6k
Veh 2.5AI 7.5AI 3CC 2.5AI 7.5AI 3CC
p-p70s6k
Veh 2.5AI 7.5AI 3CC 2.5AI 7.5AI 3CC
p-p70s6k
Veh 2.5AI 7.5AI 3CC 2.5AI 7.5AI 3CC
p-p70s6k
Veh 2.5AI 7.5AI 3CC 2.5AI 7.5AI 3CC
p-p70s6k
Veh 2.5AI 7.5AI 3CC 2.5AI 7.5AI 3CC
p-p70s6k
Veh 2.5AI 7.5AI 3CC 2.5AI 7.5AI 3CC

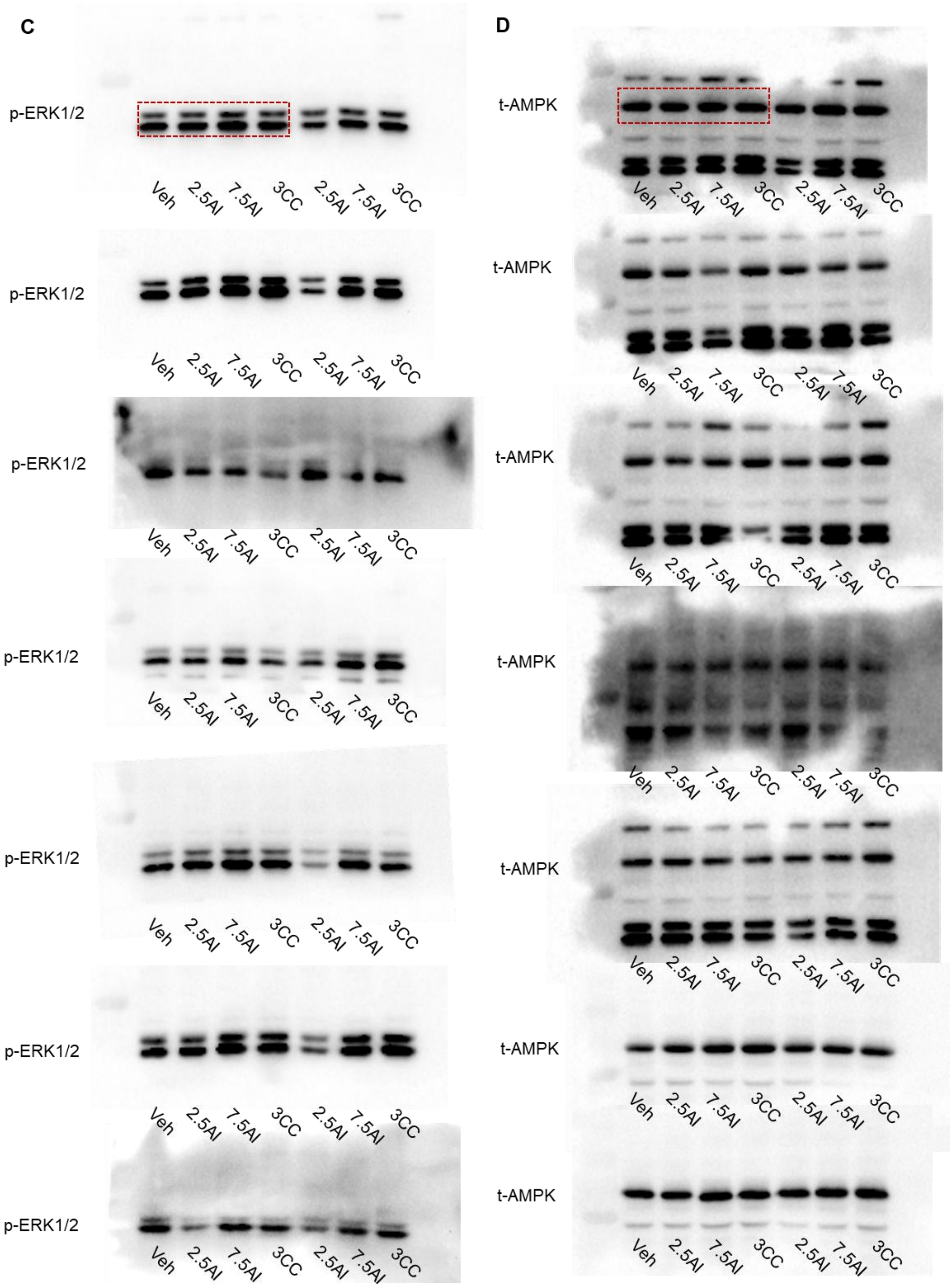

C
p-ERK1/2
Veh 2.5AI 7.5AI 3CC 2.5AI 7.5AI 3CC
p-ERK1/2
Veh 2.5AI 7.5AI 3CC 2.5AI 7.5AI 3CC
p-ERK1/2
Veh 2.5AI 7.5AI 3CC 2.5AI 7.5AI 3CC
p-ERK1/2
Veh 2.5AI 7.5AI 3CC 2.5AI 7.5AI 3CC
p-ERK1/2
Veh 2.5AI 7.5AI 3CC 2.5AI 7.5AI 3CC
p-ERK1/2
Veh 2.5AI 7.5AI 3CC 2.5AI 7.5AI 3CC
p-ERK1/2
Veh 2.5AI 7.5AI 3CC 2.5AI 7.5AI 3CC
D
t-AMPK
Veh 2.5AI 7.5AI 3CC 2.5AI 7.5AI 3CC
t-AMPK
Veh 2.5AI 7.5AI 3CC 2.5AI 7.5AI 3CC
t-AMPK
Veh 2.5AI 7.5AI 3CC 2.5AI 7.5AI 3CC
t-AMPK
Veh 2.5AI 7.5AI 3CC 2.5AI 7.5AI 3CC
t-AMPK
Veh 2.5AI 7.5AI 3CC 2.5AI 7.5AI 3CC
t-AMPK
Veh 2.5AI 7.5AI 3CC 2.5AI 7.5AI 3CC
t-AMPK
Veh 2.5AI 7.5AI 3CC 2.5AI 7.5AI 3CC

E

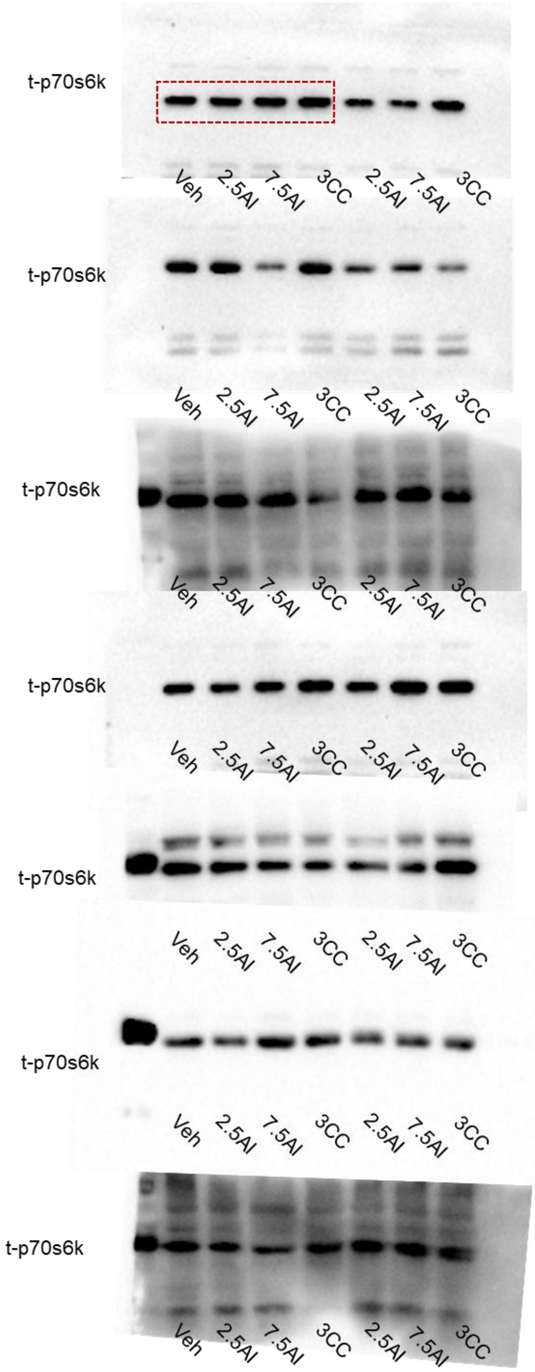
t-p70s6k
Veh
2.5AI
7.5AI
3CC
2.5AI
7.5AI
3CC
t-p70s6k
t-p70s6k
t-p70s6k
t-p70s6k
t-p70s6k
t-p70s6k

F

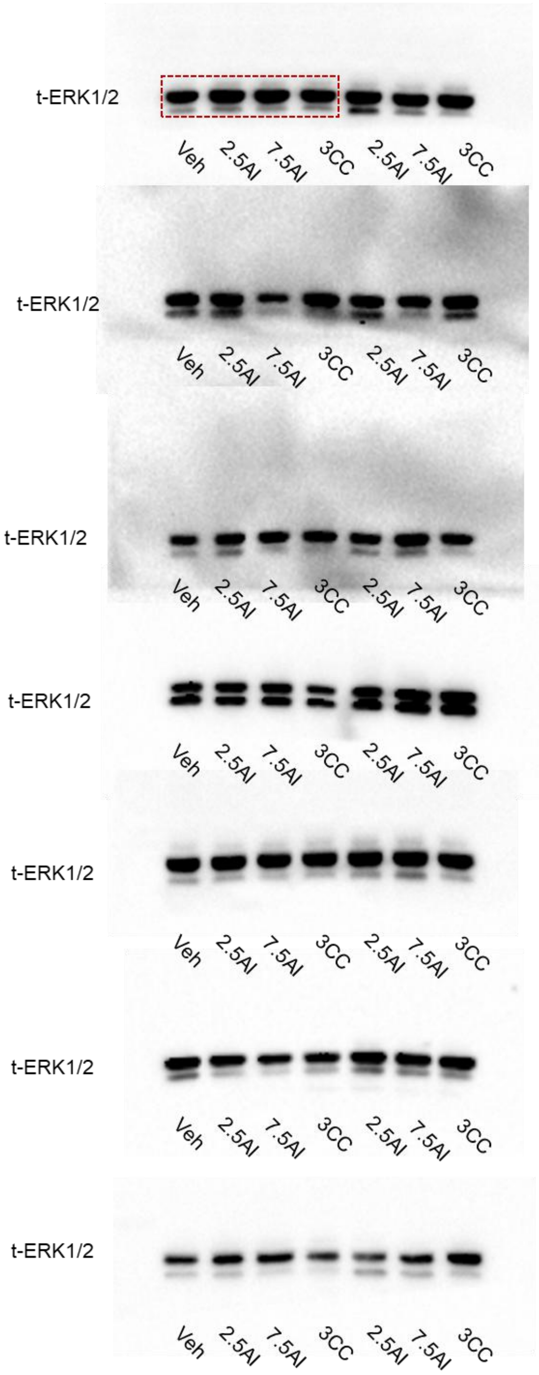
t-ERK1/2
Veh
2.5AI
7.5AI
3CC
2.5AI
7.5AI
3CC
t-ERK1/2
t-ERK1/2
t-ERK1/2
t-ERK1/2
t-ERK1/2
t-ERK1/2

G

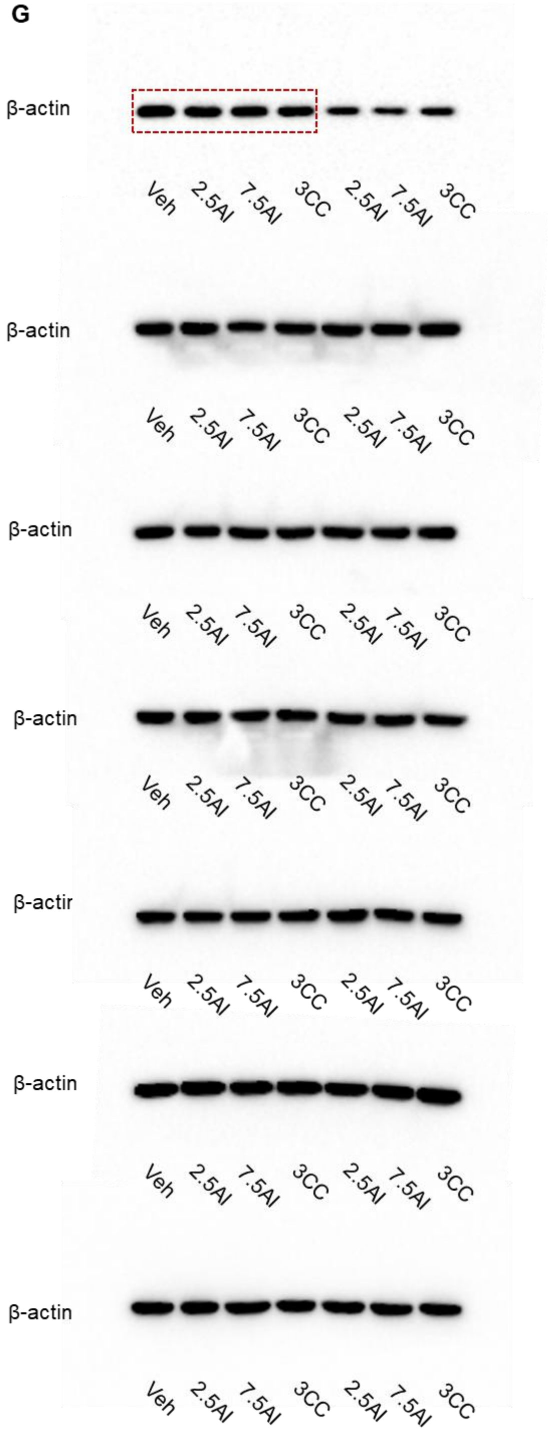

Supplementary Figure 8. Original full-length images of Western blots of phosphorylated AMPK (A), phosphorylated p70s6k (B), phosphorylated ERK1/2 (C), total AMPK (D), total p70s6k (E), total ERK1/2 (F), and β-actin (G) provided in Fig. 4F in the main text and all replicates. Veh, vehicle; 2.5AI, 2.5μg AICAR; 7.5AI, 7.5μg AICAR; 3CC, 3μg comp C.
